# Supplementary material for: Cell Identity Switching Regulated by Retinoic Acid Signaling Maintains Homogeneous Segments in the Hindbrain
Source: Dev Cell. 2018 Jun 4;45(5):606–620.e3. doi: 10.1016/j.devcel.2018.04.003 (PMC5988564; doi:10.1016/j.devcel.2018.04.003)
Supplement: Document S1. Figures S1–S4 and Table S1 [file mmc1.pdf]

Developmental Cell, Volume *45*

Supplemental Information

Cell Identity Switching Regulated by Retinoic Acid Signaling Maintains Homogeneous Segments in the Hindbrain

Megan Addison, Qiling Xu, Jordi Cayuso, and David G. Wilkinson

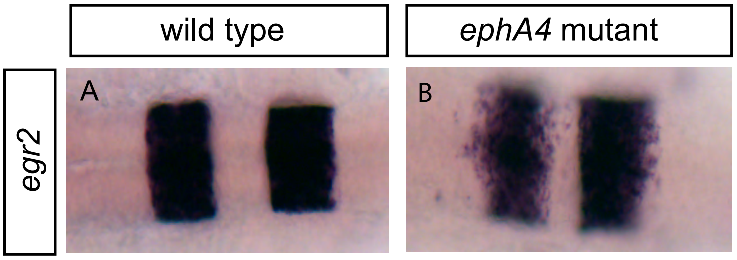

Figure S1, related to Figure 3. Analysis of *ephA4* mutant embryos
(A, B): An *ephA4* mutant has disrupted sharpening of the r2/r3, r3/r4 and r5/r6 borders. MO-mediated knockdown disrupts *ephA4* function since the same phenotype occurs in *ephA4* morphant embryos (Cooke et al., 2005; Terriente et al., 2012).

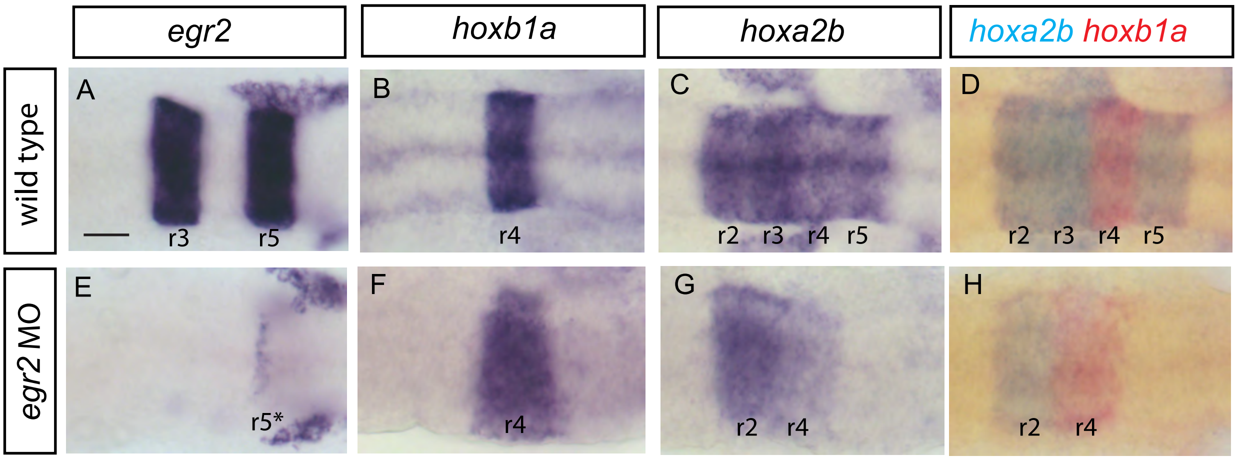

Figure S2, related to Figure 4. Analysis of *egr2* knockdown embryos
(A-H): Knockdown of *egr2a* and *egr2b* results in loss of *egr2b* expression in r3 and severe reduction of expression in r5* at 17 hpf (E) compared to control embryos (A). Following loss of r3 territory, the flanking segments still express *hox* genes that mark r2 (*hoxa2*) and r4 (*hoxb1* plus *hoxa2*): compare controls (B-D) with *egr2* knockdown embryos (F-H). A-C and E-G are single in situ hybridizations, and D, H are double in situ hybridizations, as indicated. Scale bar: 50 μm.

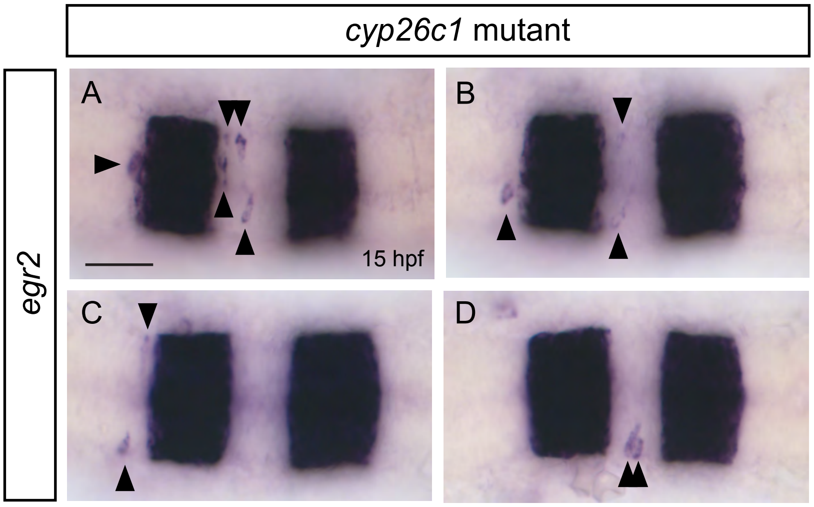

Figure S3, related to Figure 6A-E. *cyp26c1* mutant embryos have ectopic *egr2*-expressing cells
(A-D): Expression of *egr2* was analyzed in *cyp26c1* mutant embryos. Ectopic *egr2*-expressing cells were observed in r2 and r4 (arrowheads), as seen in morphants (Fig.6B). That this phenotype occurs following inactivation of *cyp26c1* alone suggests that RA levels in r2 and r4 are altered sufficiently to disrupt identity switching. Scale bar: 50 µm.

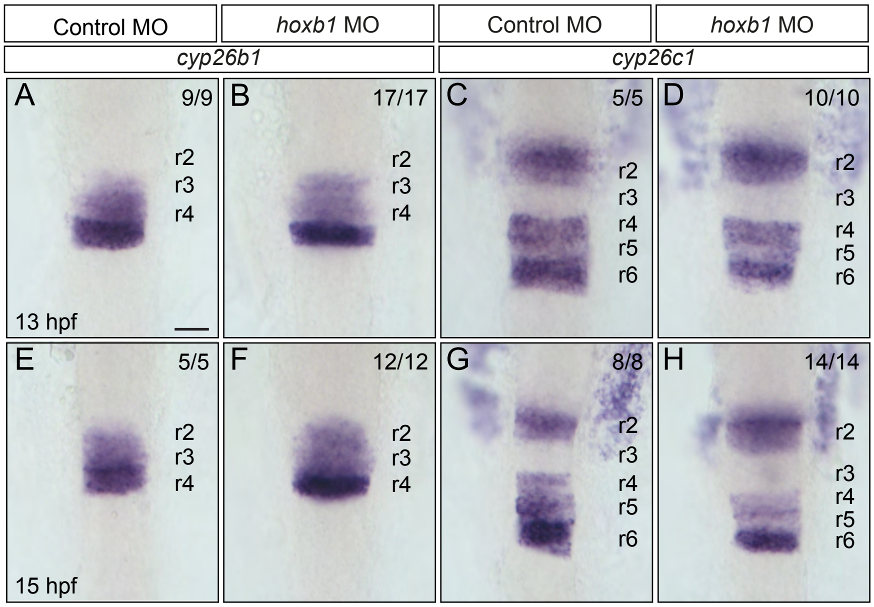

Figure S4, related to Figure 6F-H. *hoxb1* knockdown does not alter *cyp26b1* or *cyp26c1* expression
(A-H): *hoxb1a* and *hoxb1b* were knocked down and expression of *cyp26b1* and *cyp26c1* analyzed. While knockdown of *hoxb1* increases the size of r3 at the expense of r4, *cyp26b1* is still highly expressed in r4 at 13 hpf (B) and 15 hpf (E), as in control embryos (A, E). Similarly, *cyp26c1* is still expressed in r4 of *hoxb1* morphants at 13 hpf (D) and 15 hpf (H), as in control embryos (C, G). Embryos in are flat-mounted with anterior to the top. Scale bars: 50 µm.

Table S1. Related to STAR Methods section. Oligonucleotides used in the paper

Cloning oligos

**Name**	**Sequence 5'-3'**	**Target**
egr2b-attB F	GGGGACAAGTTTGTACAAAAAAGCAGGCTGGACTTCACGATGACAGCTAAAACTTTG	egr2b for Gateway pME
egr2b-attB R	GGGGACCACTTTGTACAAGAAAGCTGGGTGGTTTGAACTGGACGAGCAGATGC	egr2b for Gateway pME
egr2b-Myc Amp F	GATCGTCGACGCTGGACTTCACGATGACA	egr2b-Myc for CNE1:egr2b-Myc
egr2b-Myc Amp R	GGATCATCATCGATGGTAC	egr2b-Myc for CNE1:egr2b-Myc
mBait insertion F	AGCGCGGCTGCTGCGGTTCCAGAGGTGGATCGATCTCGAGAAGCTTGACGT	annealed
mBait insertion R	CAAGCTTCTCGAGATCGATCCACCTCTGGAACCGCAGCAGCCGCGCT	annealed
H2B-Citrine-pA F	GATCAAGCTTCTGCAGTCGACGGTACCGCCACC	H2B-Citrine-polyA
H2B-Citrine-pA R	CGCCGCGGCCGCGAATTAAAAAACCTCCCACAC	H2B-Citrine-polyA
cFos insertion F	CGATCCAGTGACGTAGGAAGTCCATCCATTCACAGCGCTTCTATAAAGGCGCCAGCTGA GGCGCCTACTACTCCAACCGCGACTGCAGCGAGCAACTA	annealed
cFos insertion R	AGCTTAGTTGCTCGCTGCAGTCGCGGTTGGAGTAGTAGGCGCCTCAGCTGGCGCCTTTATAGA AGCGCTGTGAATGGATGGACTTCCTACGTCACTGGAT	annealed

Morpholinos

**Name**	**Sequence 5'-3'**	**Target**	**Reference**	**Dose**
Standard control MO	CCTCTTACCTCAGTTACAATTTATA			4-5 ng/embryo
p53 MO	GCGCCATTGCTTTGCAAGAATTG	p53	Langheinrich et al., 2002	4 ng/embryo
egr2b MO	AGTTTTAGCTGTCATCGTGAAGTCC	egr2b	this study	4 ng/embryo
egr2a MO	CATGTGCTCCATGTTGGGAAGATTT	egr2a	this study	4 ng/embryo
ephA4 MO	AACACAAGCGCAGCCATTGGTGTC	ephA4	Cooke et al., 2005	5 ng/embryo
cyp26b1 MO	CTCGAAGAGCATGGCTGTGAACGTC	cyp26b1	Hernandez et al., 2007	4 ng/embryo

cyp26c1 MO	AAACTCGGTTATCCTCACCTTGCGC	cyp26c1	Hernandez et al., 2007	4 ng/embryo
hoxb1a MO	GGAACTGTCCATACGCAATTAA	hoxb1a	McClintock et al., 2002	4 ng/embryo
hoxb1b MO	AATTCATTGTTGACTGACCAAGCAA	hoxb1b	McClintock et al., 2002	4 ng/embryo

gRNAs

Name	Sequence 5'-3'	Target	Reference	Dose
egr2b	(GG)ATTCTGAGCTATCCAGTACGG	egr2b	this study	10 pg/embryo
mBait	GGCTGCTGCGGTTCCAGAGGTGG	mBait	this study	50 pg/embryo
cyp26c1 1	CCATGGATCCCTGCGGGAGTGGG	cyp26c1	this study	32 pg/embryo
cyp26c1 2	GGCCAGCCCATGGATCCCTGCGG	cyp26c1	this study	32 pg/embryo
ephA4	CCTGCGTGAAGCTTTCATCAGCC	ephA4	this study	32 pg/embryo

gRNA oligos

Name	Sequence 5'-3'	Target
egr2b F	TAGGATTCTGAGCTATCCAGTA	egr2b
egr2b R	AAACTACTGGATAGCTCAGAAT	egr2b
mBait F	TAGGCTGCTGCGGTTCCAGAGG	mBait
mBait R	AAACCCTCTGGAACCGCAGCAG	mBait

Target sequences for HCR detection of egr2b transcripts

Sequences 5'-3'	Target	Reference
CTGACAGCTTTCCACATGTAACGCTTCGTGCGCGCGTTCGCACAGACACAAC	egr2b	this study
ATTCTGTGAACATCCGAGCGAGTGCTTCTTAGGACTTCACGATGACAGCTAA		
ACGGGGATATGAGCACGGAGAAGCGCGCCCTCGACTTAGCCTACTCCAGCAG		
GACCAGTGCCTGACGGACCCCGGCTACCCACAGCTTACACTCCGCAGAATTT		
GCATCTATTCGGTGGACGAGCTTGCCACAACACTGCCAGCCTCTGTGACTAT		
ATAACGATTTAGGAGGACATTACGAGCAGATAAACGCAGGAGATGGCCTGAT		
TCGCGCAACCAGCTGGCCCTCGCAACCAAACTTTTACCTACATGGGAAAGTT		
CCATCGACTCCCAGTACCCGGGAAACTGGAACCCAGAGGGCGTGATCAACAT		
AGTCGCATGCTGACTGACCCAAACGCACTTTGTTTGAATATTTAGGCCAATT		
CAAACTAAACTTTCAAGGAGGTGTCTAACATCACTATTTTCTCCCATCGTCG		
ACTGTTATACATGTATTTGTTATGAAGTGACTAACGCGGGTCAATAATGTCC		
CTTTGGAGAAAGCCCCTGTGAGTCTCGGTGGCTTTGTGCACCCTCTTGCCGA		
TCTCATATCCTCCGCCATCCTACTCCTCTCCAAAGCCAAACGCCGACTCTGG		
TGTTCCCTATAATCCCGGACTACGCCGGCTTTTTCCAACCTCCGTGCCAGAG		
AATTACCGCCTCCTCTAACCCCCCTGAACACTATCAGGAACTTCACGCTAGG		
AGTTCGCGAGGAGCGACGAAAGAAAGAGACACACCAAAATCCACCTGCGACA		
AAGAGCGAAAGTCCTCCTCGTCGTCCACAGGAGTGTCCAGCTCAGAGCGCGG		
TCGCCACGAGCATCTGCTCGTCCAGTTCAAACCAGTGAACTTTCAACTGGAC		
TGCAATATCCAACACTATGTAGGCAATACAATAATGCACCGTTCGTGTTTTA		
AAAACTGGGTGGACTGATGAGGTGAAATCAAACCACAGTGCCAAACATGGAC		
