# Supplementary material for: Cell Identity Switching Regulated by Retinoic Acid Signaling Maintains Homogeneous Segments in the Hindbrain
Source: Dev Cell. 2018 Jun 4;45(5):606–620.e3. doi: 10.1016/j.devcel.2018.04.003 (PMC5988564; doi:10.1016/j.devcel.2018.04.003)
Supplement: Document S2. Article plus Supplemental Information [file mmc2.pdf]

Article

Developmental Cell

Cell Identity Switching Regulated by Retinoic Acid Signaling Maintains Homogeneous Segments in the Hindbrain

Graphical Abstract

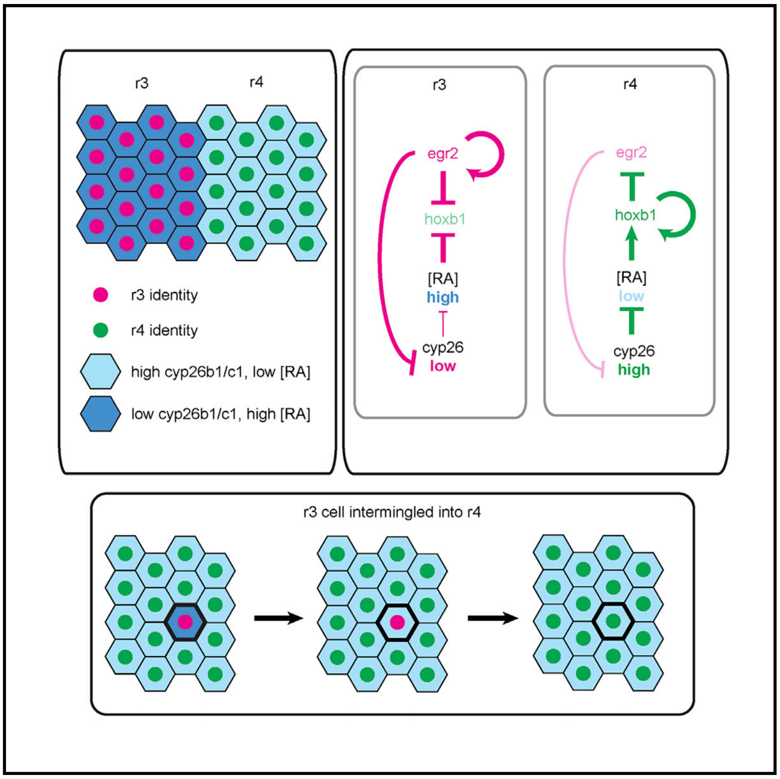

Authors

Megan Addison, Qiling Xu, Jordi Cayuso, David G. Wilkinson

Correspondence

david.wilkinson@crick.ac.uk

In Brief

Addison et al. uncover how segments within the hindbrain maintain a homogeneous identity despite intermingling of cells at early stages. Cells that intermingle into an adjacent segment switch identity since they encounter a different level of retinoic acid signaling from their new neighbors.

Highlights

- Some cells intermingle between hindbrain segments and switch identity
- Identity is influenced by the number of neighbors with same or different identity
- Switching is mediated by feedback between segment identity and retinoid signaling
- Community retinoid signaling enables maintenance of homogeneous segments

Addison et al., 2018, Developmental Cell *45*, 606–620
June 4, 2018 © 2018 The Author(s). Published by Elsevier Inc.
https://doi.org/10.1016/j.devcel.2018.04.003

Cell Identity Switching Regulated by Retinoic Acid Signaling Maintains Homogeneous Segments in the Hindbrain

Megan Addison,[1] Qiling Xu,[1] Jordi Cayuso,[1] and David G. Wilkinson[1,2,*]
[1]Neural Development Laboratory, The Francis Crick Institute, 1 Midland Road, London NW1 1AT, UK
[2]Lead Contact
*Correspondence: david.wilkinson@crick.ac.uk
https://doi.org/10.1016/j.devcel.2018.04.003

SUMMARY

The patterning of tissues to form subdivisions with distinct and homogeneous regional identity is potentially disrupted by cell intermingling. Transplantation studies suggest that homogeneous segmental identity in the hindbrain is maintained by identity switching of cells that intermingle into another segment. We show that switching occurs during normal development and is mediated by feedback between segment identity and the retinoic acid degrading enzymes, *cyp26b1* and *cyp26c1*. *egr2*, which specifies the segmental identity of rhombomeres r3 and r5, underlies the lower expression level of *cyp26b1* and *cyp26c1* in r3 and r5 compared with r2, r4, and r6. Consequently, r3 or r5 cells that intermingle into adjacent segments encounter cells with higher *cyp26b1*/c1 expression, which we find is required for downregulation of *egr2b* expression. Furthermore, *egr2b* expression is regulated in r2, r4, and r6 by non-autonomous mechanisms that depend upon the number of neighbors that express *egr2b*. These findings reveal that a community regulation of retinoid signaling maintains homogeneous segmental identity.

INTRODUCTION

The complex organization of the adult body arises during development by formation of distinct tissues at different locations, many of which are then further subdivided into domains with a specific regional identity. Such regionalization occurs along the anterior-posterior (A-P) axis of the vertebrate central nervous system to form subdivisions that are demarcated by sharp borders. A-P patterning of the neural epithelium is achieved through graded cell signaling, mediated by retinoic acid (RA) and members of the Fgf and Wnt families (Tuazon and Mullins, 2015), which regulates the spatial expression of transcription factors that specify regional identity (Parker and Krumlauf, 2017; Tumpel et al., 2009). However, at early stages the borders between different subdivisions are ragged and there can be overlapping expression of transcription factors that confer different identities. This imprecision is likely due in part to variability in the formation and interpretation of gradients of signals. In addition, the proliferation and intercalation of cells during tissue growth and morphogenesis can potentially scramble the pattern by causing intermingling of cells between adjacent regions. This raises the questions of how, despite these challenges, a sharp border forms at the interface of subdivisions, and each subdivision acquires a homogeneous regional identity. Insights into underlying mechanisms have come from studies of the vertebrate hindbrain.

The hindbrain is subdivided into seven segments, termed rhombomeres (r1–r7), which underlie the organization and A-P specification of neurons and branchial neural crest cells (Lumsden and Krumlauf, 1996). Regionalization of the hindbrain is established by graded Fgf and RA signaling, which regulates the spatial expression of a network of transcription factors that underlie the formation and A-P identity of hindbrain segments, including Egr2 (Krox20), MafB, and Hox family members (Tumpel et al., 2009). Initially, there is some overlap at borders between *hoxb1* expression in r4, and *egr2* expression in r3 and r5, which is resolved bdevcel_4183_gr4_4c.eps - y mutual repression such that cells express one or the other transcription factor (Giudicelli et al., 2001; Labalette et al., 2015; Zhang et al., 2012). The borders of *egr2* expression in r3 and r5 are ragged when first detected, and then progressively become sharp and straight (Cooke and Moens, 2002; Irving et al., 1996; Kemp et al., 2009). This sharpening is driven by signaling between segmentally expressed Eph receptors and ephrins that segregates cells and prevents intermingling across borders (Cooke et al., 2001, 2005; Kemp et al., 2009; Xu et al., 1995, 1999), potentially through regulation of cell adhesion, tension, and/or repulsion (Calzolari et al., 2014; Cayuso et al., 2015; Fagotto et al., 2014; Taylor et al., 2017). Computer simulations suggest that cell segregation and the resolution of cell identity have synergistic roles in border sharpening (Wang et al., 2017).

A further mechanism required to establish segments with homogeneous identity was suggested by the results of clonal analyses in the chick hindbrain. Once rhombomeres are seen at the morphological level, intermingling of cells is restricted across segment borders, but the progeny of individual cells labeled at earlier stages can contribute to adjacent segments (Fraser et al., 1990). The finding that some intermingling occurs between hindbrain segments implies that cells that move into another segment acquire an identity in accordance with their new A-P location. Direct evidence for an ability of hindbrain cells to switch A-P identity has come from transplantation

 © 2018 The Author(s). Published by Elsevier Inc.
This is an open access article under the CC BY license (http://creativecommons.org/licenses/by/4.0/).

experiments in mouse and zebrafish embryos. It was found that when single cells are transplanted between hindbrain segments, they downregulate markers of their site of origin and switch to the identity of their new location (Kemp et al., 2009; Schilling et al., 2001; Trainor and Krumlauf, 2000). In zebrafish, cells can switch identity at early stages of segmentation (11.5 hr post fertilization [hpf]), but this plasticity progressively decreases at later stages (14–16.5 hpf) (Schilling et al., 2001). In contrast to single cells, groups of cells transplanted between segments maintain their original identity, suggestive of a community regulation of cell identity (Schilling et al., 2001; Trainor and Krumlauf, 2000). Such community effects have been found in other contexts to be mediated by positive feedback between transcription factors and intercellular signals that regulate cell identity (Bolouri and Davidson, 2010; Buckingham, 2003; Cossu et al., 1995; Gurdon, 1988; Standley et al., 2001). Through non-autonomous induction of transcription factor expression, this feedback promotes a homogeneous identity within a field of cells (Bolouri and Davidson, 2010). Interestingly, mosaic overexpression of *egr2* in the chick hindbrain induces *egr2* expression in neighboring cells (Giudicelli et al., 2001), but the molecular basis of this non-autonomous induction is not known.

The findings from transplantation experiments have led to the idea that cell identity switching could act in parallel with cell segregation to establish sharp and homogeneous segments (Cooke and Moens, 2002; Pasini and Wilkinson, 2002). However, it is unclear to what extent intermingling of cells between segments occurs during normal development. *egr2* has a key role in hindbrain segmentation through specification of r3 and r5 identity (Schneider-Maunoury et al., 1993; Voiculescu et al., 2001) and is a direct transcriptional regulator of *ephA4* (Theil et al., 1998), which underlies cell segregation (Cooke et al., 2005; Xu et al., 1995, 1999). It is therefore likely that intermingling between segments is confined to the time period before there has been sufficient upregulation of EphA4 to drive cell segregation. Consistent with findings in chick (Fraser et al., 1990), some isolated cells expressing *egr2* or *egr2*-cre reporter are detected in even-numbered segments in the mouse hindbrain (Irving et al., 1996; Voiculescu et al., 2001). However, recent work has suggested that there is no intermingling between hindbrain segments in zebrafish, and therefore cell identity switching does not occur (Calzolari et al., 2014). In this study, tracking of cells in time-lapse movies from 11 hpf did not detect intermingling between segments, and fluorescent reporter expression driven downstream of *egr2* was not detected in any cells in adjacent segments (Calzolari et al., 2014). However, interpretation of these findings may be limited by timing of the analyses, as mechanisms that restrict cell intermingling may already be in place by 11 hpf and prior to detectable expression of the transgenic reporters.

We set out to analyze the role and mechanisms of cell identity switching in establishment of homogeneous segmental identity. By using genome modification to create an early reporter of *egr2* expression, we show that cell intermingling and identity switching occurs during hindbrain segmentation in zebrafish. *egr2* expression is regulated by a combination of A-P location and non-autonomous mechanisms that depend upon the number of neighbors that express *egr2*. We uncover a crucial role of RA-degrading enzymes, *cyp26b1* and *cyp26c1*, which we show are regulated by *egr2* and are required for identity switching of r3 and r5 cells that intermingle into adjacent segments. These findings reveal that coupling between segment identity and retinoid signaling enables homogeneous segmental identity to be maintained despite intermingling of cells.

RESULTS

Cell Intermingling and Identity Switching Occurs in the Zebrafish Hindbrain

In zebrafish, *egr2* is upregulated in prospective r3 and then r5, starting at 10–11 hpf respectively, and the initially ragged borders of *egr2* gene expression are sharpened over a 2 hr period (Cooke and Moens, 2002; Kemp et al., 2009; Zhang et al., 2012). Some ectopic *egr2*-expressing cells are detected in even-numbered segments, which potentially could segregate back to r3 or r5 or switch identity. To create an early-expressed reporter of cells with r3 or r5 identity, we used an enhancer trap strategy in which *H2B-Citrine* with a minimal promoter was inserted upstream of the *egr2b* gene by CRISPR/Cas9-mediated recombination (Figure 1A). This was found to drive reporter expression in r3 and r5. To maximize reporter expression, we created a line homozygous for *egr2b:H2B-Citrine*. *In situ* hybridization reveals that *citrine* and *egr2b* transcripts are concurrently expressed in r3 and r5 from the earliest time that *egr2b* expression is detected (Figures 1B–1O). Importantly, this analysis reveals that insertion of the reporter gene has not blocked expression of *egr2b*. Comparison of time-lapse movies with *in situ* hybridization data reveals that citrine fluorescence is first seen ~1 hr after detection of *egr2* transcripts.

Due to the high stability of H2B fusions with fluorescent reporters (Brennand et al., 2007; Ninov et al., 2012), H2B-Citrine will perdure in any cells that have intermingled and downregulated *egr2b* transcripts. Analysis of time-lapse movies reveals the presence of some isolated cells with Citrine fluorescence within even-numbered segments, which have not segregated back to r3 or r5 by 16 hpf (Figure 1). When these cells are back-tracked, they are found to already be surrounded by non-expressing cells at the earliest time point at which Citrine fluorescence can be detected (Figures 1P–1S). This suggests that intermingling into adjacent segments occurs at early stages, during convergent-extension movements in which there is extensive intercalation of cells (Kimmel et al., 1994). Since *egr2* expression is being upregulated during this period of convergent extension (Figures 1B–1E), the 1 hr delay in accumulating sufficient Citrine protein for direct detection may limit the ability to detect cells that have intermingled and then downregulated *egr2b* transcripts, which have a half-life of ~30 min (Bouchoucha et al., 2013).

Based on these findings, we increased sensitivity of detection by immunostaining for Citrine protein, and found that at 17 hpf there are 2–6 isolated Citrine-expressing cells per even-numbered segment (Figures 2A and 2C). As predicted from previous studies, knockdown of EphA4 increases cell intermingling between segments, with 8–13 cells expressing Citrine reporter per even-numbered segment (Figures 2B and 2C). To determine whether ectopic Citrine-expressing cells have maintained or changed identity, we carried out *in situ* hybridization for *egr2b* and *hoxb1a* transcripts. We found that Citrine-expressing cells in even-numbered segments do not express *egr2b*, and when

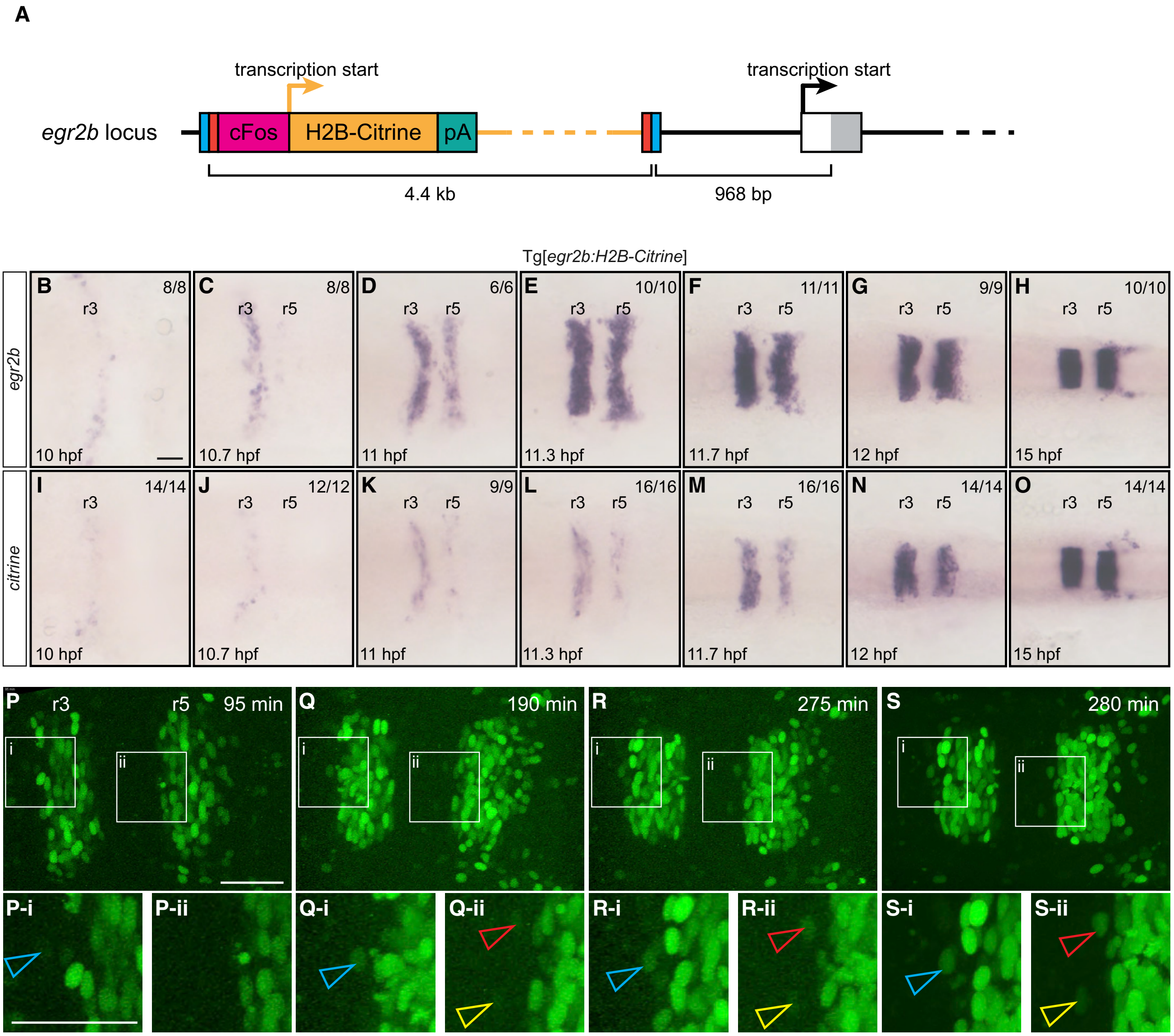

Figure 1. Generation of *egr2b* Gene Trap Expressing H2B-Citrine

(A) CRISPR-mediated insertion of a donor construct with *cFos* minimal promoter and H2B-Citrine upstream of the transcriptional start site of *egr2b* to generate the Tg[*egr2b:H2B-Citrine*] line.

(B–O) *In situ* hybridization to detect *egr2b* (B–H) and *citrine* (I–O) transcripts in Tg[*egr2b:H2B-Citrine*] embryos from 10 to 15 hpf. Embryos are flat-mounted with anterior to the left.

(P–S) Selected frames from a time-lapse movie of a Tg[*egr2b:H2B-Citrine*] embryo acquired from 12 hpf (t = 0 min) for 280 min. A higher resolution image was captured at the final time point (S). Below each panel is a magnified view of the indicated areas (i, ii), with arrowheads pointing at three examples of H2B-Citrine-expressing cells that are ectopic at the final time point. When first detected, these cells are already surrounded by non-expressing cells. Most of the H2B-Citrine-expressing cells seen posterior to r5 are *egr2*-expressing neural crest cells. Scale bars: 50 μm.

present in r4 express *hoxb1a*, and have therefore switched to r4 identity (Figure 2D).

Cell Organization Influences Non-autonomous Induction of *egr2b* Expression

Our findings reveal that at early stages there is some intermingling of cells expressing *egr2b* into adjacent segments, and these ectopic cells switch identity. This raises the question of how cell identity switching is regulated. Since the initial pattern of segmental identities is established by graded signaling (Hernandez et al., 2007; White et al., 2007; White and Schilling, 2008), one potential mechanism for switching is that cells respond to the amount of signal present at their new location. However, this model does not explain why groups of transplanted cells do not switch identity (Schilling et al., 2001; Trainor and Krumlauf, 2000). Experiments in chick embryos suggest that *egr2* could be a component of a community regulation of cell identity since forced mosaic expression of *egr2* in r2, r4, and r6

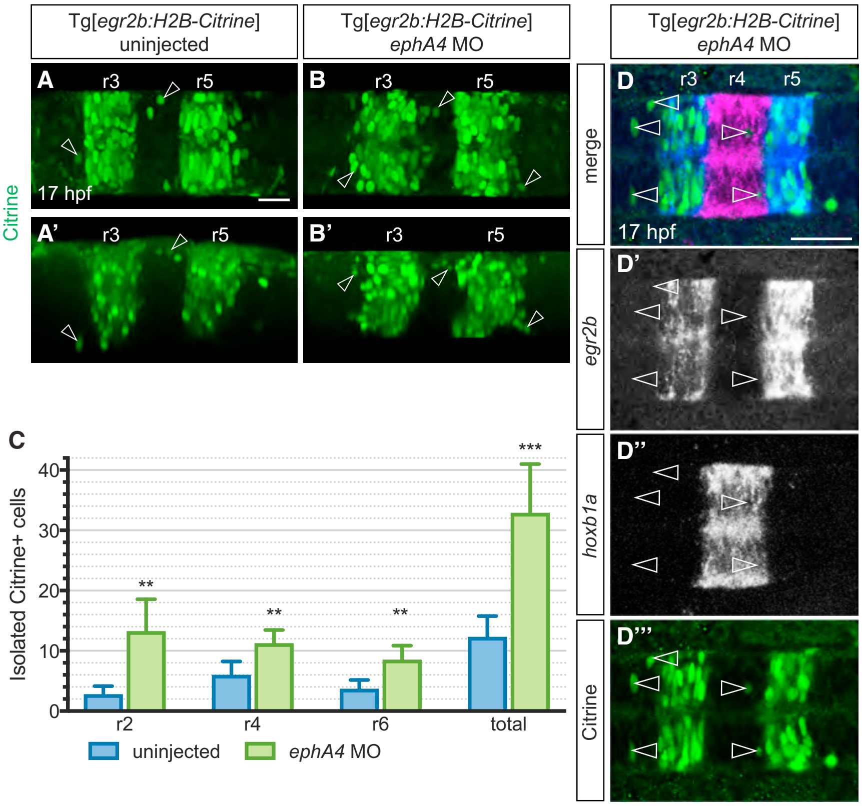

Figure 2. Cell Identity Switching of Ectopic *egr2:H2B-Citrine*-Expressing Cells

(A and B) Maximum intensity projections of typical uninjected (A) and *ephA4* morphant (B) Tg[*egr2b:H2B-Citrine*] embryos at 17 hpf, with H2B-Citrine protein detected using anti-GFP antibody. Lateral views of the same embryos are shown in (A′) and (B′). Examples of Citrine-positive cells outside r3 and r5 are indicated by arrowheads.

(C) Quantification of isolated ectopic Citrine-positive cells in uninjected and *ephA4* morphant embryos. Mean numbers with 95% confidence intervals are shown. Asterisks indicate statistical significance determined by Welsh's t test: r2, p = 0.0014; r4, p = 0.0017; r6, p = 0.0016; total isolated ectopic cells in r2, r4, and r6, p = 0.0002. n = 18 embryos (uninjected), n = 11 embryos (*ephA4* morphants).

(D) Double *in situ* hybridization reveals that isolated Citrine-expressing cells (green in D‴, arrowheads in D, D″) in r2, r4, and r6 of *ephA4* morphant Tg[*egr2b:H2B-Citrine*] embryos have downregulated *egr2b* transcripts (blue in D, grayscale in D′; 105/121 cells). Citrine-expressing cells located in r4 have upregulated *hoxb1a* (magenta in D, scale in D″). Single slices from a confocal z stack are shown. Scale bars: 50 μm.

induces *egr2* expression in adjacent cells (Giudicelli et al., 2001). We therefore analyzed whether *egr2* expression is influenced by interactions with neighboring cells.

We first tested whether non-autonomous induction of *egr2* expression occurs in the zebrafish hindbrain. To achieve sustained expression of myc-tagged Egr2b, we used a *pax3* regulatory element (CNE1) that drives gene expression in the dorsal neural epithelium (Moore et al., 2013). Injection of this construct into zebrafish embryos led to mosaic ectopic expression of *egr2b*. We found that, at 12.5 hpf, cells ectopically expressing *egr2b* are scattered, and that endogenous *egr2b* is upregulated in adjacent cells in r2, r4, and r6 (Figure 3A). In contrast, by 17 hpf the *egr2b*-overexpressing cells have segregated to the borders of even-numbered segments, and non-autonomous induction of *egr2b* no longer occurs (Figure 3B). One potential explanation for this lack of non-autonomous induction is that it is influenced by the proportion of neighboring cells that express *egr2b*. Another possibility is that cells no longer respond to signaling from *egr2*-expressing cells as they have reduced plasticity at the later stage (Schilling et al., 2001). Since segregation of *egr2b*-expressing cells is mediated by EphA4 (Cooke et al., 2005; Theil et al., 1998; Xu et al., 1995), they can be maintained in a dispersed state by simultaneous knockdown of *ephA4*. We found that, in this situation, non-autonomous induction of *egr2b* occurs at 17 hpf (Figure 3C).

To further test the relationship between cell organization and non-autonomous *egr2* induction, we created embryos that have mosaic ectopic expression of the endogenous *egr2* gene. We took advantage of the finding that *egr2* gene regulation involves an autoregulatory element that maintains expression following an initial pulse of *egr2* expression (Bouchoucha et al., 2013; Chomette et al., 2006). We established a transgenic line with heat-shock-regulated *egr2b* (*hs-egr2b*) and used this to induce an early pulse of *egr2b* expression, which leads to ectopic expression of endogenous *egr2b* in even-numbered segments (Figures 3D and 3E) (Bouchoucha et al., 2013; Chomette et al., 2006). We transplanted labeled wild-type cells into the *hs-egr2* line, induced *egr2b* expression, and analyzed whether there is non-autonomous induction of *egr2* expression in wild-type cells present in even-numbered segments (Figure 3F). We found that wild-type cells express *egr2b* if intermingled with *hs-egr2b* cells (Figure 3G), but groups of wild-type cells do not upregulate *egr2b* (Figure 3H). Taken together, these findings suggest that non-autonomous induction of *egr2b* expression in even-numbered segments depends upon having a sufficient number of neighbors that are expressing *egr2b*.

In view of these findings, we wondered whether cells downregulate *egr2b* expression when surrounded by non-expressing cells, as occurs when they intermingle into adjacent segments. Since autoregulation is required to sustain expression, knockdown of *egr2b* leads to loss of *egr2b* expression in r3 and to a lesser extent in r5 by 17 hpf, leading to depletion of r3 and r5 territory, whereas markers of r2 and r4 identity are still expressed (Figure S2) (Bouchoucha et al., 2013; Chomette et al., 2006). By transplanting wild-type cells into *egr2b* morphant embryos (Figure 4A), we created embryos in which only some cells are competent to maintain *egr2b* expression. We found that even small groups of wild-type cells present at the border of r2 and

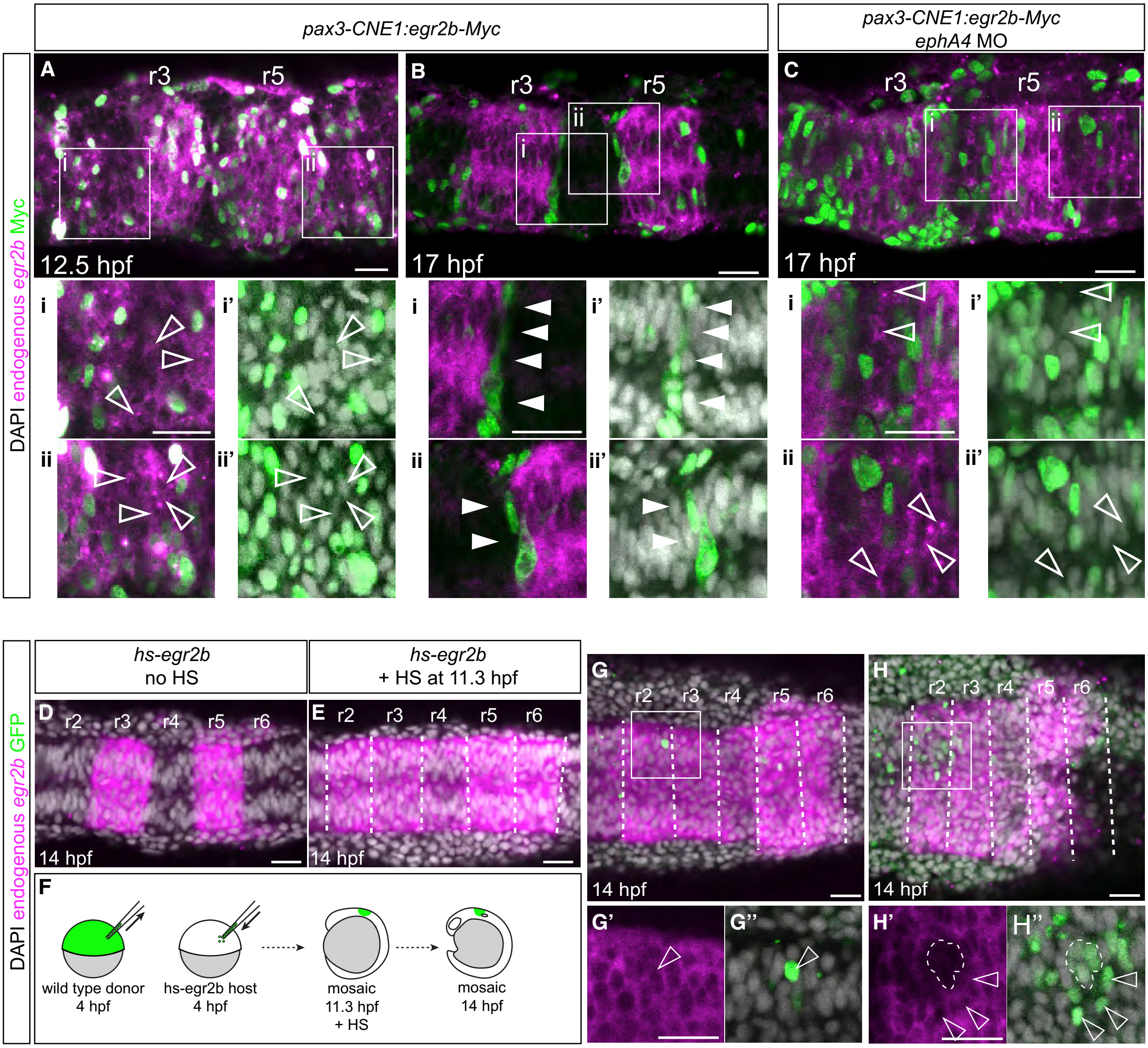

Figure 3. Relationship between Cell Organization and Non-autonomous Induction of *egr2* Gene Expression

(A–C) Embryos injected with *CNE1:egr2b-Myc* and fixed at various time points to detect endogenous *egr2b* transcripts. At 12 hpf (A), endogenous *egr2b* gene expression (magenta) is upregulated in cells overexpressing Egr2b-Myc (green nuclei) and non-autonomous upregulation of *egr2b* is also observed in adjacent cells (hollow arrowheads, Ai and Aii). DAPI staining (grayscale) is shown in (Ai′) and (Aii′). By 17 hpf (B), Egr2b-Myc-expressing cells become segregated to the borders of r3 and r5, and non-autonomous upregulation of *egr2b* is no longer observed in adjacent cells (Bi and Bii, solid arrowheads). Knockdown of *ephA4* (see also Figure S1) in embryos injected with *CNE1:egr2b-Myc* (C) causes cells overexpressing Egr2b-Myc to remain dispersed throughout the neuroepithelium at 17 hpf and non-autonomous upregulation of *egr2b* is now observed (Ci and Cii, hollow arrowheads).

(D–H) Transient expression of *egr2b* by heat shock of Tg[*hsp70:egr2b-Myc*] embryos (*hs-egr2b*) at 11.3 hpf induces upregulation of endogenous *egr2b* in r2, r4, and r6 (E); compare with control embryo shown in (D). H2B-GFP labeled wild-type donor cells were transplanted into *hs-egr2b* embryos at 4 hpf, which were heat shocked at 11.3 hpf to induce widespread expression of *egr2b* (F). Wild-type donor cells in r2 upregulate endogenous *egr2b* non-autonomously when isolated or dispersed (G, H, hollow arrowheads) but not when clustered together, indicated by the region encircled by white dashed line in (H). White dashed lines in (E), (G), and (H) indicate presumptive rhombomere borders. Single slices from a confocal z stack are shown. Scale bars: 50 μm.

r4 are able to express *egr2b* (r3* in Figure 4B). As a further test, we prevented clustering of *egr2*-expressing cells by carrying out analogous experiments with *ephA4* morphant cells transplanted into *egr2* morphant embryos (Figure 4C). We found that *egr2b*-expressing cells were still present, even when organized as single cells at the border of r2 and r4 (Figure 4D). Thus, *egr2b* expression can be maintained in cells at the normal A-P location of r3 when surrounded by cells which are not expressing *egr2b*. This suggests that A-P patterning of cell identity is dominant over community signaling.

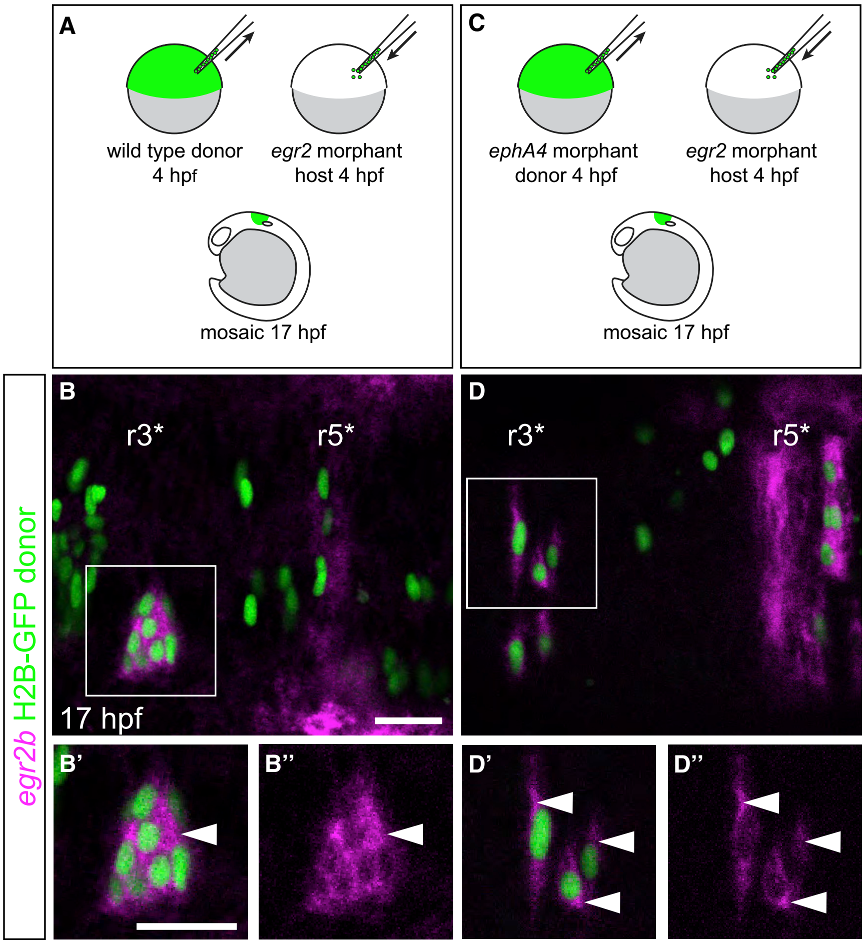

Figure 4. Effect of Decreasing the Number of *egr2b*-Expressing Cells

(A and B) H2B-GFP-labeled wild-type donor cells were transplanted into *egr2a* plus *egr2b* morphant hosts (A). Since autoregulation is required to maintain *egr2* expression (Figure S2), only the transplanted cells can express *egr2* in r3 at this stage. Wild-type donor cells at the position of r3 (denoted r3*) in *egr2* morphants form a small cluster and maintain *egr2b* expression at 17 hpf (B, white arrowhead).

(C and D) H2B-GFP-labeled *ephA4* morphant donor cells were transplanted into *egr2* morphant hosts (C). *ephA4* morphant donor cells no longer cluster in r3* but still maintain *egr2b* expression (D, white arrowheads). Single slices from a confocal z stack are shown. Scale bars: 50 μm.

Egr2b Regulates the Segmental Expression Level of *cyp26b1* and *cyp26c1*

Our findings raise the question of how *egr2* expression can occur in single cells at the correct A-P location but is downregulated in isolated cells present in even-numbered segments, whereas groups of cells can maintain segmental identity in an ectopic location. Previous studies have shown that a high posterior to low anterior gradient of RA has a key role in establishing segmental identity in the hindbrain (Tumpel et al., 2009; White and Schilling, 2008). A gradient of RA is created by a counter-gradient of the RA-degrading enzyme, *cyp26a1*, which is regulated by RA and Fgf signaling (White et al., 2007). Two other family members, *cyp26b1* and *cyp26c1*, are expressed in dynamic segmental patterns in the hindbrain, and unlike *cyp26a1* are not direct targets of RA signaling (Gu et al., 2005; Hernandez et al., 2007; Sirbu et al., 2005; Zhao et al., 2005). This dynamic regulation of *cyp26b1* and *cyp26c1* expression may also establish a gradient of RA that underlies A-P patterning (Hernandez et al., 2007; Sirbu et al., 2005). Since knockdown of *cyp26b1* or *cyp26c1* only leads to changes in segment identity when combined with loss of *cyp26a1* function (Hernandez et al., 2007), these *cyp26* family members have overlapping roles in regulating RA levels. We speculated that *cyp26b1* and *cyp26c1* may have a further role in which they are regulated by segment identity genes, and act in a homeostatic feedback mechanism that maintains an RA level appropriate for the A-P position. Furthermore, by acting as a sink for RA, Cyp26 enzymes can have non-autonomous effects by influencing RA levels in adjacent cells (Rydeen et al., 2015; Rydeen and Waxman, 2014; White et al., 2007). We therefore wondered whether there is feedback between *egr2* and *cyp26* genes that contributes to regulation of the segmental identity of cells.

To test this idea, we carried out loss and gain of function experiments to determine whether *cyp26b1* and *cyp26c1* expression is regulated by *egr2*. *cyp26b1* is expressed in r2-r4, at lower levels in r2 and r3 than in r4 (Figures 5A and 5E), and *cyp26c1* is expressed in r2, r4, and r6, with lower expression in r3 and r5 (Figures 5C and 5G). Thus collectively, *cyp26b1* and *cyp26c1* are expressed at lower levels in r3 and r5 than in r2, r4, and r6. We found that, following *egr2* knockdown, *cyp26b1* expression in r3 increases to the same level as in r4 (Figures 5B and 5F), and *cyp26c1* expression increases in r3 and r5 (Figures 5D and 5H). In gain of function experiments, we induced an early pulse of *egr2b* expression using the *hs-egr2b* transgenic line, such that endogenous *egr2b* is upregulated throughout the hindbrain (Figure 3E). We found that, in this situation, *cyp26b1* and *cyp26c1* expression is lower in r4, and now at the same level as in r3, and that *cyp26c1* expression also decreases in r2 and r6 (Figures 5I–5P). The results of these loss and gain of function experiments reveal that *egr2b* underlies the lower level of *cyp26b1* and *cyp26c1* expression in r3 and r5 compared with r2, r4, and r6.

Feedback Occurs between *egr2b* Expression and RA Signaling

The above findings suggest a model in which the repression of *cyp26b1* and *cyp26c1* expression by *egr2* leads to an increased level of RA, which in turn enables maintenance of *egr2* expression. To test this, we analyzed whether RA signaling contributes to the regulation of endogenous *egr2b* expression in r2, r4, and

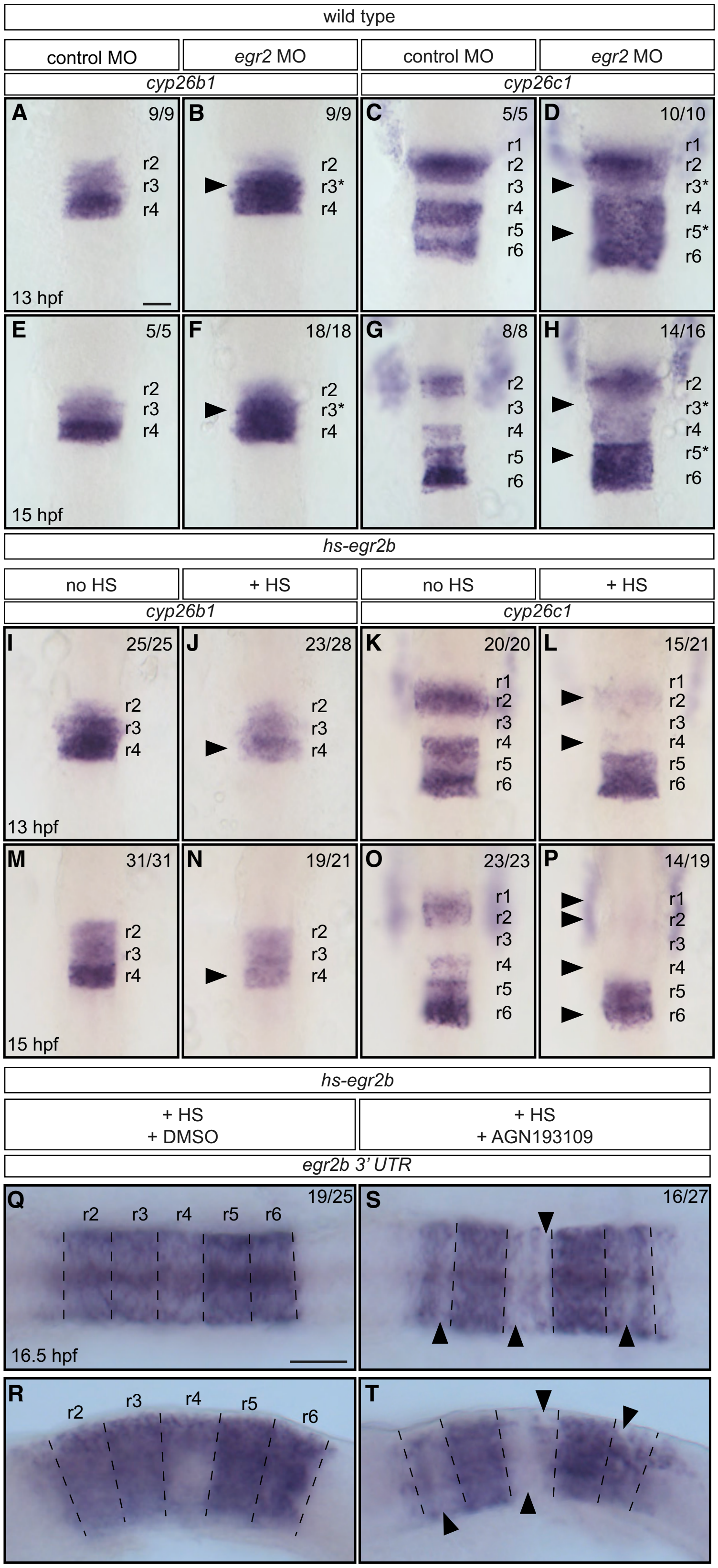

Figure 5. Relationships between *egr2*, *cyp26b1*, *cyp26c1*, and RA Signaling

(A–H) Effect of *egr2a* plus *egr2b* knockdown (*egr2* morpholino oligonucleotides [MO]) on *cyp26b1* and *cyp26c1* expression. *egr2* knockdown increases the level of *cyp26b1* expression in r3 (black arrowhead) at 13 hpf (B) and 15 hpf (F), and of *cyp26c1* in r3 and r5 (black arrowheads) at 13 hpf (D) and 15 hpf (H) compared with control embryos (A, C, E and G). r3* and r5* in (B), (D), (F), and (H) refer to the region specified as r3 or r5 in the presence of *egr2*.

(I–P) Expression of *cyp26b1* and *cyp26c1* in control embryos and after induction of widespread *egr2* expression by heat shock of *hs-egr2b* embryos at 11.3 hpf. Widespread expression of *egr2b* leads to reduction of *cyp26b1* expression in r4 (black arrowhead) at 13 hpf (J), and 15 hpf (N) compared with control embryos (I and M). Similarly, *cyp26c1* expression is reduced in r2, r4, and, to a lesser extent, in r6 (black arrowheads) at 13 hpf (L) and 15 hpf (P) in comparison with control embryos (K and O).

(Q–T) *hs-egr2b* embryos subjected to heat shock at 11.3 hpf in the presence (S and T) or absence (Q and R) of the pan-RAR antagonist AGN193109. Endogenous *egr2b* expression was analyzed at 16.5 hpf. In DMSO-treated control embryos, endogenous *egr2b* is expressed in r2, r4, and r6, but to a lesser extent in ventral r4 (Q and R). In AGN193109-treated embryos, expression of endogenous *egr2b* in r2, r4, and r6 is more heterogeneous and there are patches of cells that do not express endogenous *egr2b* (black arrowheads in S and T).

Embryos in (A)–(P) are flat-mounted with anterior to the top; embryos in (Q) and (S) are flat-mounted with anterior to the left; embryos in (R) and (T) are side-mounted with anterior to the left. Dashed lines in (Q)–(T) indicate presumptive rhombomere borders. Scale bars: 50 μm.

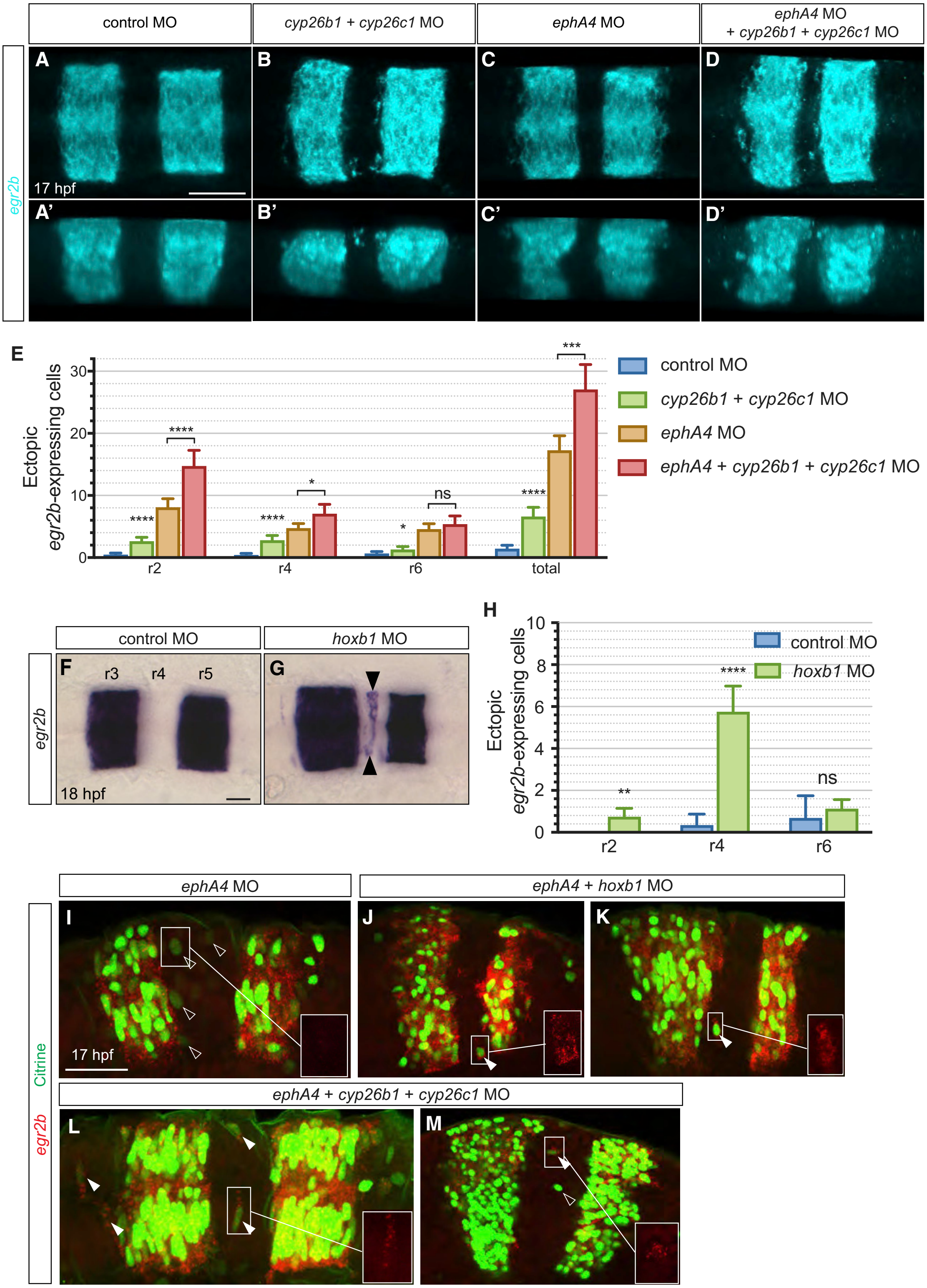

Figure 6. *cyp26b1*, *cyp26c1*, and *hoxb1* Knockdown Increases Ectopic *egr2*-Expressing Cells

(A–D) *egr2b* expression in 17 hpf embryos in which *cyp26b1* and *cyp26c1* were knocked down, compared with control morphants. Dorsal (A–D) and lateral (A′–D′) maximum intensity projections are shown. Knockdown of *cyp26b1* and *cyp26c1* causes an increased number of *egr2b*-expressing cells in r2, r4 and r6 (B and B′) compared with control embryos (A and A′); see also Figure S3. Combined knockdown of *cyp26b1*, *cyp26c1*, and *ephA4* increases the number of *egr2b* expressing cells in r2 and r4 (D and D′) compared with *ephA4* morphant embryos (C and C′) and *cyp26b1* plus *cyp26c1* knockdown alone (B and B′).

(legend continued on next page)

r6 that is induced by a pulse of *hs-egr2b* expression. In control embryos, this leads to sustained *egr2* expression throughout the hindbrain due to transcriptional autoregulation of the *egr2* gene (Bouchoucha et al., 2013; Chomette et al., 2006), which at 16.5 hpf is uniform, except for low levels in ventral r4 (Figures 5Q and 5R). We used a chemical blocker to inhibit retinoic acid receptor (RAR) following the pulse of *egr2* expression and found that this does not alter expression in r3 or r5, but has a striking effect in even-numbered segments at 16.5 hpf. We found that there is lower expression of *egr2b* in even-numbered segments compared with controls, with the strongest decrease in r4 (Figures 5S and 5T). Interestingly, following RAR inhibition, the level of *egr2b* expression is no longer homogeneous in r2, r4, and r6, and is organized in stripes of high and low expression. These findings suggest that, in addition to direct transcriptional autoregulation, ectopic *egr2b* expression in r2, r4, and r6 is promoted by positive feedback through downregulation of *cyp26b1* and *cyp26c1* and increased RA signaling.

cyp26b1 and *cyp26c1* Contribute to Cell Identity Switching

The finding that low RA signaling antagonizes the maintenance of *egr2* expression in r2, r4, and r6 suggests a potential basis for cell identity switching. r3 or r5 cells that intermingle into even-numbered segments encounter territory with higher *cyp26b1* plus *cyp26c1* expression and thus lower RA levels, and this may contribute to downregulation of *egr2* expression. To test this, we analyzed the effect of knocking down *cyp26b1* and *cyp26c1*. We found that *cyp26b1* plus *cyp26c1* knockdown leads to an increased number of *egr2b*-expressing cells in r2, r4, and r6 (Figures 6A, 6B, and 6E), though with only a small increase in r6. This phenotype is also seen in embryos that are null mutants for *cyp26c1* (Figure S3). As a further test, we increased the amount of cell intermingling between segments by carrying out analogous experiments with simultaneous knockdown of *ephA4*. We found that, when *cyp26b1* and *cyp26c1* knockdown is combined with the loss of *ephA4* function, there is a 4-fold increase in the number of ectopic *egr2b*-expressing cells in r2 and r4, but not in r6, compared with *cyp26b1* plus *cyp26c1* knockdown only (Figures 6C–6E). These findings support that identity switching of *egr2b*-expressing cells present in r2 or r4 requires RA signaling regulated by *cyp26b1* and *cyp26c1*.

Our findings raise the question of how retinoid signaling affects *egr2* expression in even-numbered segments, since dissection of gene regulatory elements has not found a direct input of RA (Bouchoucha et al., 2013; Chomette et al., 2006). One potential explanation is that RA signaling in even-numbered segments promotes expression of a repressor of *egr2* gene expression. *hoxb1* is a good candidate to mediate such a mechanism in r4 since it is directly regulated by retinoid signaling (Marshall et al., 1994; Studer et al., 1994; Tumpel et al., 2009) and represses *egr2* expression (Giudicelli et al., 2001; Labalette et al., 2015; Zhang et al., 2012). We therefore carried out knockdown of *hoxb1a* and *hoxb1b*. We found that *hoxb1* knockdown does not alter *cyp26b1* or *cyp26c1* expression (Figure S4) but leads to an increase in the number of isolated *egr2*-expressing cells in r4 (Figures 6F–6H).

Taken together, these findings suggest that *cyp26b1*, *cyp26c1*, and *hoxb1* mediate identity switching of r3 and r5 cells that have intermingled into adjacent segments. To directly test this, we carried out knockdowns in the *egr2:H2B-Citrine* line followed by detection of citrine protein and *egr2* transcripts. As shown previously (Figure 2), *egr2* transcripts are not detected in citrine-expressing cells that have intermingled into r4 following *ephA4* knockdown (Figure 6I). In contrast, *egr2* transcripts are detected in ectopic Citrine-expressing cells following knockdown of *hoxb1* (Figures 6J and 6K) or *cyp26b1* plus *cyp26c1* (Figures 6L and 6M).

DISCUSSION

Transplantation experiments in the hindbrain revealed plasticity in cell fate specification, which could enable homogeneous segmental identity to be maintained despite intermingling of cells across segment borders. To study whether cell intermingling and identity switching occurs during normal development in zebrafish, we created an early reporter of *egr2b* expression by insertion of *H2B-Citrine* into the *egr2b* gene locus. We find that cell identity switching occurs during hindbrain segmentation and show that this is regulated by coupling between segment identity and retinoid signaling. Our findings suggest a model in which r3 and r5 cells that intermingle into adjacent segments switch identity since they encounter cells with a higher expression level of *cyp26b1* and *cyp26c1* (Figure 7).

Cell Intermingling and Identity Switching

Cell lineage analysis in the chick hindbrain found that the clonal progeny of cells labeled before morphological boundaries are seen can contribute to adjacent segments, but when cells are labeled after boundary formation clones are confined to

(E) Mean number of *egr2b*-expressing cells in even-numbered segments with 95% confidence intervals. Since blocking identity switching will favor segregation of cells that have initially intermingled, we counted *egr2*-expressing cells adjacent to r3 and r5 as well as isolated cells. Asterisks indicate statistical significance determined by Welsh's t test. For *cyp26b1* and *cyp26c1* morphants (n = 40) compared with control morphants (n = 22): r2, $p < 0.0001$; r4, $p < 0.0001$; r6, $p = 0.038$; all misplaced *egr2b*-expressing cells, $p < 0.0001$. For *ephA4*, *cyp26b1*, and *cyp26c1* triple morphants (n = 21) compared with *ephA4* morphants (n = 33): r2, $p < 0.0001$; r4, $p = 0.01$; r6, $p = 0.31$; total, $p = 0.0001$.

(F–H) Knockdown of *hoxb1a* and *hoxb1b* (*hoxb1* MO) increases the number of *egr2b*-expressing cells in r4 (black arrowheads) at 18 hpf (G) compared with control embryos (F). *hoxb1* knockdown does not affect *cyp26b1* or *cyp26c1* expression (Figure S4). (H) Mean number of ectopic *egr2b*-expressing cells with 95% confidence intervals. Asterisks indicate statistical significance determined by Welsh's t test: r2, $p = 0.001$; r4, $p < 0.0001$; r6, $p = 0.38$. Control embryos, n = 6; *hoxb1* morphants, n = 26.

(I–M) Knockdowns in the *egr2b:H2B-Citrine* line followed by detection of Citrine protein with anti-GFP antibody (green) and *egr2b* transcripts (red). The box in the bottom right of each panel is a magnified view of the *egr2b* transcript signal in the indicated area. Following knockdown of *ephA4* alone to increase cell intermingling, ectopic Citrine-expressing cells do not express detectable *egr2b* transcripts (I). *egr2b* transcripts are detected in ectopic Citrine-expressing cells when *ephA4* knockdown is combined with *hoxb1* (J and K) or *cyp26b1* plus *cyp26c1* knockdown (L and M). Empty arrowheads indicate Citrine-expressing cells lacking detectable *egr2b* transcripts, and filled arrowheads Citrine-expressing cells that express *egr2b* transcripts. Scale bars: 50 μm.

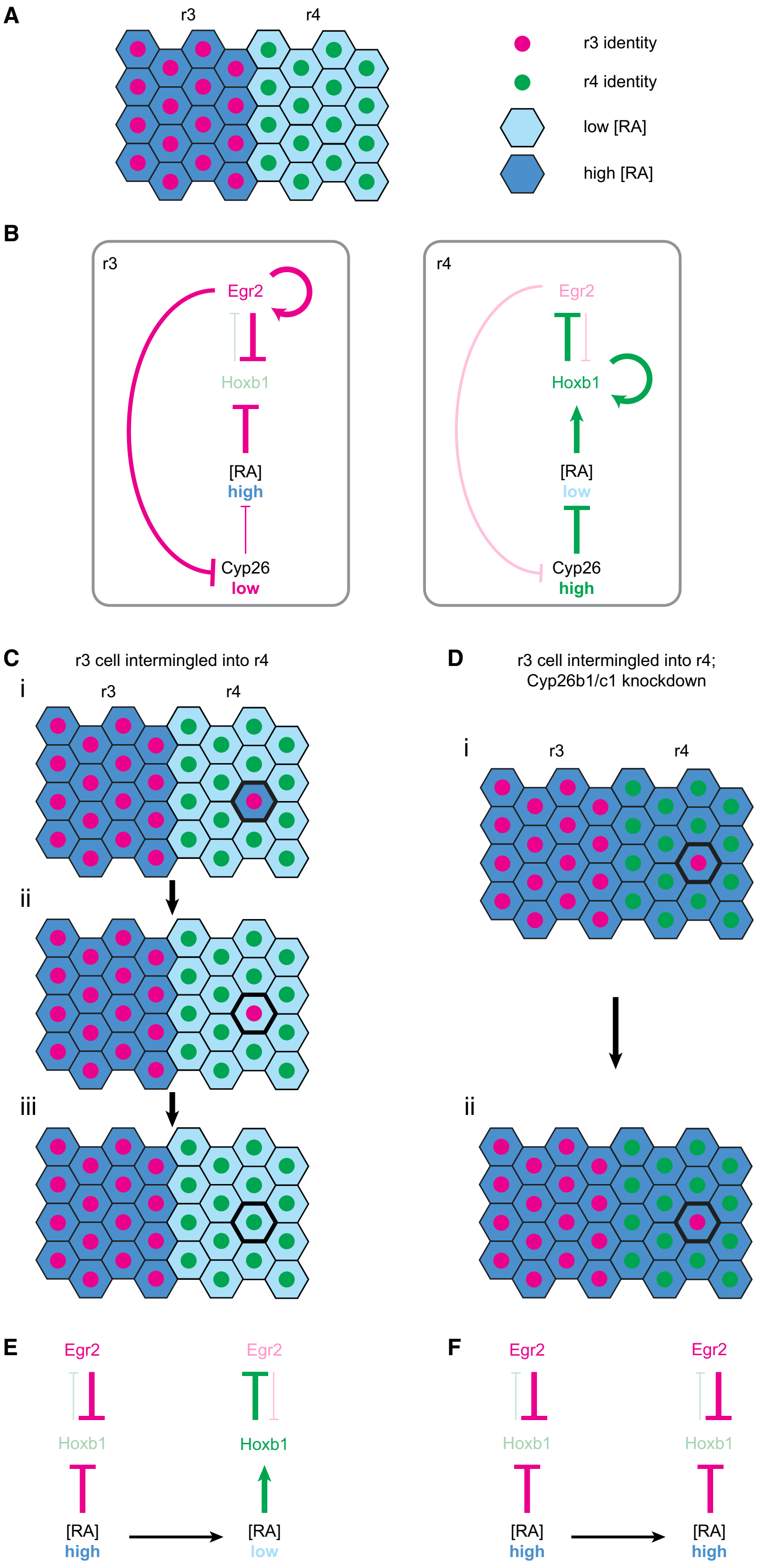

Figure 7. Model of *cyp26b1*, *cyp26c1*, and *hoxb1* in Cell Identity Switching

(A) Segmental regulation of low *cyp26b1* and *cyp26c1* expression in r3 (magenta) and high expression in r4 (green) maintains high [RA] in r3 and low [RA] in r4.

(B) In r3, *egr2* represses *cyp26b1* and *cyp26c1* expression, which in turn keeps RA levels high, repressing *hoxb1* expression. Egr2 also directly autoregulates its own expression and represses *hoxb1* expression. In r4, high *cyp26b1* and *cyp26c1* expression maintains a low level of RA, permitting expression of *hoxb1*. Hoxb1 autoregulates its own expression and represses expression of *egr2*.

(C and E) When an r3 cell intermingles into r4, the surrounding cells have high *cyp26b1* and *cyp26c1* expression, which reduces the level of RA within the ectopic r3 cell. This promotes upregulation of *hoxb1* in the ectopic r3 cell, which represses *egr2* expression, causing the r3 cell to change identity to r4.

(D and F) When an r3 cell intermingles into r4, but *cyp26b1* and *cyp26c1* are knocked down, the surrounding r4 cells no longer have high *cyp26b1/c1* expression, so RA levels remain high in the ectopic r3 cell. Consequently, *egr2* is not downregulated in the ectopic r3 cell (F).

single segments (Fraser et al., 1990). These findings can be explained by the hierarchical relationships between genes that regulate segmental identity and cell segregation. The *egr2* gene specifies r3 and r5 identity (Schneider-Maunoury et al., 1993; Voiculescu et al., 2001), which is coupled to A-P specification through reciprocal interactions with *hox* genes (Sham et al., 1993; Tumpel et al., 2009), and is also a direct transcriptional regulator of *ephA4* (Theil et al., 1998). Signaling between *ephA4* in r3/r5 and ephrins expressed in r2/r4/r6 underlies morphological boundary formation and the restriction of cell intermingling across borders (Cooke et al., 2005; Sela-Donenfeld et al., 2009; Xu et al., 1995). It is therefore likely that intermingling between segments is largely confined to the period before EphA4 has been sufficiently upregulated to mediate cell segregation. In zebrafish, this period is also when convergent extension of the neural epithelium occurs, which through cell intercalation drives extensive intermingling along the A-P axis (Kimmel et al., 1994). Based on these considerations, detection of intermingling between segments requires analysis with early reporters, and can explain why it was not observed in a study that used reporters expressed one step downstream of *egr2* (Calzolari et al., 2014). This latter study used either an *egr2* autoregulatory element driving GFP or a Gal4 gene trap in *egr2b* that drives expression of UAS-mCherry, and reporter expression was first seen in r5 at 12.8 hpf, ~2 hr after *egr2b* transcripts are detected. Since sharp borders have formed by this stage, it is likely that intermingling between segments has been restricted by the time that reporter expression is detected. The reporter line that we created enables direct detection of H2B-Citrine ~1 hr after *egr2b* expression, and sensitivity was further increased by immunodetection of citrine protein.

We find isolated cells expressing H2B-Citrine reporter in even-numbered segments, which do not have a detectable level of *egr2b* transcripts, and express *hoxb1a* when present in r4. Thus, r3 and r5 cells that have intermingled into adjacent segments have switched identity to that of their new neighbors. One potential mechanism for switching is that, at the new location, cells respond to graded signals that specify A-P position. However, this does not account for the finding that groups of cells retain their original identity when transplanted to another segment (Schilling et al., 2001; Trainor and Krumlauf, 2000). The finding that forced mosaic expression of *egr2* in even-numbered segments can non-autonomously induce *egr2* expression (Giudicelli et al., 2001) suggests that this transcription factor regulates community signaling. We find that non-autonomous induction occurs when cells with forced *egr2* expression are intermingled with r2, r4, or r6 cells, but not when *egr2*-expressing and non-expressing cells are segregated. The induction of *egr2* expression in r2, r4, and r6 cells thus depends upon the number of *egr2*-expressing cells that they contact, suggesting that segmental identity is influenced by short-range interactions in which the majority wins.

Roles of *cyp26b1* and *cyp26c1*

Previous studies have shown that the A-P specification of hindbrain segments is mediated by a posterior to anterior gradient of RA generated by Cyp26 enzymes. There is a distinct expression and regulation of different *cyp26* family members in the hindbrain. *cyp26a1* is expressed in an anterior to posterior gradient that forms at early stages and is directly regulated by RA and FGF signaling (White et al., 2007). In contrast, *cyp26b1* and *cyp26c1* are not directly regulated by RA, and are expressed in dynamic segmental patterns (Hernandez et al., 2007; Sirbu et al., 2005). Since *cyp26b1* and *cyp26c1* knockdown only has a strong effect on A-P patterning when combined with *cyp26a1* loss of function, these family members are thought to have parallel roles. Our findings provide evidence for a distinct role of *cyp26b1* and *cyp26c1*, in which they underlie identity switching of *egr2*-expressing cells that have intermingled into even-numbered segments.

We show that *egr2* underlies the lower level of *cyp26b1* and *cyp26c1* expression in r3 and r5 compared with r2, r4, and r6. Since Egr2 is a transcriptional activator, the repression of *cyp26b1* and *cyp26c1* expression may be indirect, but there is evidence that in some contexts Egr2 can act as a repressor (Desmazieres et al., 2009; Mager et al., 2008; Sevetson et al., 2000). It is currently not known how *cyp26b1* or *cyp26c1* expression is regulated in r2, r4, and r6. Although we find that *hoxb1* knockdown has no effect on *cyp26b1* or *cyp26c1* expression, it is possible that other *hox* genes are involved. The expression pattern of *cyp26b1* and *cyp26c1* suggests that, following initial patterning of the hindbrain by graded RA, a more complex pattern is formed with higher RA levels in odd- compared with even-numbered segments. The currently available transcriptional reporter lines are only sensitive enough to detect the high RA levels present in the spinal cord (reviewed by Schilling et al., 2016). Techniques for direct detection of RA have revealed a gradient in the hindbrain (Shimozono et al., 2013; Sosnik et al., 2016), but, as the RA levels detected are noisy at the spatial resolution of hindbrain segments, it is unclear whether there is a continuously decreasing gradient or a more complex pattern.

A consequence of coupling *cyp26b1* and *cyp26c1* expression levels to segment identity (Figures 7A and 7B) is that, when r3 or r5 cells intermingle into adjacent segments, they will initially retain low expression of *cyp26b1* and *cyp26c1*, and are moving into territory with higher *cyp26b1/c1* expression. Previous work has shown that, in addition to a strong cell autonomous effect on RA levels, Cyp26 enzymes have a weak non-autonomous effect on adjacent cells, presumably by acting as a local sink for RA (Rydeen et al., 2015; Rydeen and Waxman, 2014; White et al., 2007). This predicts that, when single cells intermingle into an adjacent segment, the amount of RA they experience can be influenced by the level of Cyp26 enzymes in their neighbors. We find that knockdown of *cyp26b1* plus *cyp26c1* leads to an increase in the number of ectopic *egr2*-expressing cells in r2 and r4, although not in r6. Furthermore, there is a greater number of ectopic *egr2*-expressing cells when intermingling is increased by simultaneous loss of ephA4 function. Thus, *cyp26b1* and *cyp26c1* enable the identity switching of r3 and r5 cells that have intermingled into adjacent segments. The reason why they are required in r2 and r4, but not r6, is unclear, but may relate to the anterior to posterior progression of *cyp26b1* and *cyp26c1* expression, which is upregulated in r2–r4 at 10 hpf, and in r6 at 11 hpf (Gu et al., 2005; Hernandez et al., 2007). This suggests that another mechanism acts to mediate identity switching in r6 during early stages when intermingling between segments occurs.

Further evidence for how *egr2b* is regulated when expressed in r2, r4, or r6 came from use of a heat shock inducible line to induce transient *egr2b* expression, which due to autoregulation is then maintained throughout the hindbrain. Since ectopic *egr2b* expression downregulates *cyp26b1* and *cyp26c1* expression in r2, r4, and r6, this is predicted to increase RA signaling to a similar level as occurs in r3 and r5. We find that inhibition of RAR function disrupts the maintenance of *egr2b* expression in even-numbered segments, leading to a heterogeneous mixture of expressing and non-expressing cells. Taken together, these findings reveal that low RA signaling (high *cyp26b1/c1* or inhibition of RAR) promotes downregulation of *egr2b* expression in even-numbered segments, and that high RA (knockdown of *cyp26b1/c1*, or overexpression of *egr2*, which represses *cyp26b1* and *cyp26c1*) enables maintenance of *egr2* expression. We therefore propose that an *egr2*-expressing cell that intermingles into an adjacent segments is surrounded by cells with a higher level of *cyp26b1/c1*, which non-autonomously decreases RA levels in the *egr2*-expressing cell and leads to identity switching (Figure 7C). In contrast, such identity switching does not occur when an *egr2*-expressing cell intermingles into territory in which *cyp26b1/c1* levels have been lowered by gene knockdown (Figure 7D).

Relationship between RA Signaling and *egr2* Expression

Since dissection of gene regulatory elements has not found any direct input of RA into regulation of *egr2* (Bouchoucha et al., 2013; Chomette et al., 2006), our findings raise the question of how changes to RA levels affect *egr2* gene expression in even-numbered segments. We tested a role of *hoxb1*, since it represses *egr2* expression (Giudicelli et al., 2001; Labalette et al., 2015; Zhang et al., 2012) and is directly upregulated in r4 and repressed in r3 and r5 by retinoid signaling (Lee and Skromne, 2014; Marshall et al., 1994; Studer et al., 1994; Tumpel et al., 2009). Consistent with previous studies (Labalette et al., 2015; Zigman et al., 2014), we find that knockdown of *hoxb1* genes leads to an increase in the number of *egr2*-expressing cells in r4. We therefore propose that lower RA levels maintained by *cyp26b1* and *cyp26c1* in r4 enable upregulation of *hoxb1* expression in r3 and r5 cells that have intermingled, which in turn represses *egr2b* expression (Figures 7C and 7E). Following knockdown of *cyp26b1* and *cyp26c1*, there is a higher level of RA in r4, which does not promote upregulation of *hoxb1*, and consequently *egr2b* expression is maintained (Figures 7D and 7F).

Distinct Regulation of *egr2* at Normal and Ectopic Locations

Whereas single ectopic cells downregulate *egr2*, we find that isolated cells can express *egr2* when present at the normal A-P position of r3. This suggests that A-P regulation of *egr2* expression is dominant over the proposed community signaling. Similarly, inhibition of RAR at late stages leads to downregulation of ectopic *egr2* expression in r2, r4, and r6 but does not disrupt *egr2* expression in r3 and r5. These findings are consistent with the mechanisms that regulate *egr2* gene expression. Due to the combinatorial input of Fgf signaling and Hox and Nlz transcription factors, an initiator element that regulates *egr2* expression is strongly activated in r3 but has weak activity in r2 and r4 (Labalette et al., 2015). This generates sufficient Egr2 protein in r3 to maintain and amplify subsequent expression through an autoregulatory element (Bouchoucha et al., 2013; Chomette et al., 2006). Our findings suggest that strong activation of the initiator element plus autoregulation is able to maintain *egr2* gene expression even in single r3 cells flanked by r2 and r4 cells, which can have non-autonomous effects on RA levels. In contrast, r3 or r5 cells that intermingle into adjacent segments are in territory with low activation of the initiator element, and thus the non-autonomous effects of *cyp26b1* and *cyp26c1* are able to antagonize the maintenance of *egr2* expression.

Community Regulation of Cell Identity

In classical models of community effects, a transcription factor that specifies cell identity upregulates expression of a signaling molecule that can induce expression of the transcription factor in neighboring cells (Bolouri and Davidson, 2010; Buckingham, 2003; Standley et al., 2001). Our findings provide evidence for a related mechanism, in which coupling between segment identity and the expression level of retinoid degrading enzymes sets the amount of RA signaling in a group of cells. The coupling of *cyp26b1* and *cyp26c1* expression to segment identity can account for the finding that groups of cells maintain their original identity when transplanted to another segment (Schilling et al., 2001; Trainor and Krumlauf, 2000). Consistent with this, mosaic overexpression of *cyp26c1* induced ectopic *hoxb1* gene expression when present in groups of cells but not in single cells (Lee and Skromne, 2014). Interestingly, r3 and r5 gene expression occurred in groups of cells in adjacent segments following overexpression of truncated EphA4 (Xu et al., 1995), which is predicted to greatly increase cell intermingling by both blocking Eph receptor activation and activating ephrin reverse signaling. The extent of intermingling that occurs during normal development, or even single Eph receptor knockdown, leads to isolated ectopic cells that come under the influence of the level of *cyp26b1/c1* expression and RA degradation in neighboring cells. In this mechanism, cell identity regulation depends upon how many neighbors are of the same or different type, and thus switching does not occur at borders of segregated cell populations. This may provide a more reliable mechanism for switching than responding to an RA gradient, which is not able to specify identity along the A-P axis with single-cell precision (Zhang et al., 2012).

STAR★METHODS

Detailed methods are provided in the online version of this paper and include the following:

- KEY RESOURCES TABLE
- CONTACT FOR REAGENT AND RESOURCE SHARING
- EXPERIMENTAL MODEL AND SUBJECT DETAILS
  - Maintenance of Zebrafish Strains
- METHOD DETAILS
  - Constructs and Transgenesis
  - Morpholino Injection
  - Pharmacological and Heat Shock Treatments
  - Cell Transplantation
  - *In Situ* Hybridization and Immunocytochemistry
  - Live Imaging and Analysis
- QUANTIFICATION AND STATISTICAL ANALYSIS
  - Quantification of Ectopic Cells

SUPPLEMENTAL INFORMATION

Supplemental Information includes four figures and can be found with this article online at https://doi.org/10.1016/j.devcel.2018.04.003.

ACKNOWLEDGMENTS

We thank Jean-Paul Vincent, Andrew Oates, and Greg Elgar for advice; James Briscoe and Caroline Hill for comments on the manuscript; and James Briscoe also for providing the CNE1 construct. We also thank the staff of the Biological Research Facility and the Light Microscopy Technology Platform at the Francis Crick Institute for their invaluable support. This work was supported by the Francis Crick Institute, which receives its core funding from Cancer Research UK (FC001217), the UK Medical Research Council (FC001217), and the Wellcome Trust (FC001217).

AUTHOR CONTRIBUTIONS

Conceptualization, D.G.W., M.A., and Q.X.; Methodology, D.G.W., M.A., Q.X., and J.C.; Investigation, M.A., Q.X., and J.C.; Writing – Original Draft, D.G.W.; Writing – Review & Editing, D.G.W., M.A., and Q.X.; Funding Acquisition, D.G.W.; Supervision, D.G.W.

DECLARATION OF INTERESTS

The authors declare no competing interests.

Received: December 4, 2017
Revised: March 16, 2018
Accepted: April 4, 2018
Published: May 3, 2018

REFERENCES

Balciunas, D., Wangensteen, K.J., Wilber, A., Bell, J., Geurts, A., Sivasubbu, S., Wang, X., Hackett, P.B., Largaespada, D.A., McIvor, R.S., et al. (2006). Harnessing a high cargo-capacity transposon for genetic applications in vertebrates. PLoS Genet. *2*, e169.

Berger, J., and Currie, P.D. (2013). 503unc, a small and muscle-specific zebrafish promoter. Genesis *51*, 443–447.

Bolouri, H., and Davidson, E.H. (2010). The gene regulatory network basis of the "community effect," and analysis of a sea urchin embryo example. Dev. Biol. *340*, 170–178.

Bouchoucha, Y.X., Reingruber, J., Labalette, C., Wassef, M.A., Thierion, E., Desmarquet-Trin Dinh, C., Holcman, D., Gilardi-Hebenstreit, P., and Charnay, P. (2013). Dissection of a Krox20 positive feedback loop driving cell fate choices in hindbrain patterning. Mol. Syst. Biol. *9*, 690.

Brennand, K., Huangfu, D., and Melton, D. (2007). All beta cells contribute equally to islet growth and maintenance. PLoS Biol. *5*, e163.

Buckingham, M. (2003). How the community effect orchestrates muscle differentiation. Bioessays *25*, 13–16.

Calzolari, S., Terriente, J., and Pujades, C. (2014). Cell segregation in the vertebrate hindbrain relies on actomyosin cables located at the interhombomeric boundaries. EMBO J. *33*, 686–701.

Cayuso, J., Xu, Q., and Wilkinson, D.G. (2015). Mechanisms of boundary formation by Eph receptor and ephrin signaling. Dev. Biol. *401*, 122–131.

Choi, H.M., Calvert, C.R., Husain, N., Huss, D., Barsi, J.C., Deverman, B.E., Hunter, R.C., Kato, M., Lee, S.M., Abelin, A.C., et al. (2016). Mapping a multiplexed zoo of mRNA expression. Development *143*, 3632–3633.

Chomette, D., Frain, M., Cereghini, S., Charnay, P., and Ghislain, J. (2006). Krox20 hindbrain cis-regulatory landscape: interplay between multiple long-range initiation and autoregulatory elements. Development *133*, 1253–1262.

Cooke, J., Moens, C., Roth, L., Durbin, L., Shiomi, K., Brennan, C., Kimmel, C., Wilson, S., and Holder, N. (2001). Eph signalling functions downstream of Val to regulate cell sorting and boundary formation in the caudal hindbrain. Development *128*, 571–580.

Cooke, J.E., Kemp, H.A., and Moens, C.B. (2005). EphA4 is required for cell adhesion and rhombomere-boundary formation in the zebrafish. Curr. Biol. *15*, 536–542.

Cooke, J.E., and Moens, C.B. (2002). Boundary formation in the hindbrain: Eph only it were simple. Trends Neurosci. *25*, 260–267.

Cossu, G., Kelly, R., Didonna, S., Vivarelli, E., and Buckingham, M. (1995). Myoblast differentiation during mammalian somitogenesis is dependent upon a community effect. Proc. Nat. Acad. Sci. USA *92*, 2254–2258.

Desmazieres, A., Charnay, P., and Gilardi-Hebenstreit, P. (2009). Krox20 controls the transcription of its various targets in the developing hindbrain according to multiple modes. J. Biol. Chem. *284*, 10831–10840.

Fagotto, F., Winklbauer, R., and Rohani, N. (2014). Ephrin-Eph signaling in embryonic tissue separation. Cell Adh. Migr. *8*, 308–326.

Fraser, S., Keynes, R., and Lumsden, A. (1990). Segmentation in the chick embryo hindbrain is defined by cell lineage restrictions. Nature *344*, 431–435.

Gerety, S.S., and Wilkinson, D.G. (2011). Morpholino artifacts provide pitfalls and reveal a novel role for pro-apoptotic genes in hindbrain boundary development. Dev. Biol. *350*, 279–289.

Giudicelli, F., Taillebourg, E., Charnay, P., and Gilardi-Hebenstreit, P. (2001). Krox-20 patterns the hindbrain through both cell-autonomous and non cell-autonomous mechanisms. Genes Dev. *15*, 567–580.

Gu, X.X., Xu, F., Wang, X.L., Gao, X., and Zhao, Q.S. (2005). Molecular cloning and expression of a novel CYP26 gene (cyp26d1) during zebrafish early development. Gene Expr. Patterns *5*, 733–739.

Gurdon, J.B. (1988). A community effect in animal development. Nature *336*, 772–774.

Hernandez, R.E., Putzke, A.P., Myers, J.P., Margaretha, L., and Moens, C.B. (2007). Cyp26 enzymes generate the retinoic acid response pattern necessary for hindbrain development. Development *134*, 177–187.

Hwang, W.Y., Fu, Y., Reyon, D., Maeder, M.L., Tsai, S.Q., Sander, J.D., Peterson, R.T., Yeh, J.R., and Joung, J.K. (2013). Efficient genome editing in zebrafish using a CRISPR-Cas system. Nat. Biotechnol. *31*, 227–229.

Irving, C., Nieto, M.A., DasGupta, R., Charnay, P., and Wilkinson, D.G. (1996). Progressive spatial restriction of Sek-1 and Krox-20 gene expression during hindbrain segmentation. Dev. Biol. *173*, 26–38.

Kemp, H.A., Cooke, J.E., and Moens, C.B. (2009). EphA4 and EfnB2a maintain rhombomere coherence by independently regulating intercalation of progenitor cells in the zebrafish neural keel. Dev. Biol. *327*, 313–326.

Kimelman, D., and Martin, B.L. (2012). Anterior-posterior patterning in early development: three strategies. Wiley Interdiscip. Rev. Dev. Biol. *1*, 253–266.

Kimmel, C.B., Ballard, W.W., Kimmel, S.R., Ullmann, B., and Schilling, T.F. (1995). Stages of embryonic development of the zebrafish. Dev. Dyn. *203*, 253–310.

Kimmel, C.B., Warga, R.M., and Kane, D.A. (1994). Cell cycles and clonal strings during formation of the zebrafish central nervous system. Development *120*, 265–276.

Kimura, Y., Hisano, Y., Kawahara, A., and Higashijima, S. (2014). Efficient generation of knock-in transgenic zebrafish carrying reporter/driver genes by CRISPR/Cas9-mediated genome engineering. Sci. Rep. *4*, 6545.

Kwan, K.M., Fujimoto, E., Grabher, C., Mangum, B.D., Hardy, M.E., Campbell, D.S., Parant, J.M., Yost, H.J., Kanki, J.P., and Chien, C.B. (2007). The Tol2kit: a multisite gateway-based construction kit for Tol2 transposon transgenesis constructs. Dev. Dyn. *236*, 3088–3099.

Labalette, C., Wassef, M.A., Desmarquet-Trin Dinh, C., Bouchoucha, Y.X., Le Men, J., Charnay, P., and Gilardi-Hebenstreit, P. (2015). Molecular dissection of segment formation in the developing hindbrain. Development *142*, 185–195.

Langheinrich, U., Hennen, E., Stott, G., and Vacun, G. (2002). Zebrafish as a model organism for the identification and characterization of drugs and genes affecting p53 signaling. Curr. Biol. *12*, 2023–2028.

Lauter, G., Soll, I., and Hauptmann, G. (2011). Two-color fluorescent in situ hybridization in the embryonic zebrafish brain using differential detection systems. BMC Dev. Biol. *11*, 43.

Lee, K., and Skromne, I. (2014). Retinoic acid regulates size, pattern and alignment of tissues at the head-trunk transition. Development *141*, 4375–4384.

Lumsden, A., and Krumlauf, R. (1996). Patterning the vertebrate neuraxis. Science *274*, 1109–1115.

Mager, G.M., Ward, R.M., Srinivasan, R., Jang, S.W., Wrabetz, L., and Svaren, J. (2008). Active gene repression by the Egr2/NAB complex during peripheral nerve myelination. J. Biol. Chem. *283*, 18187–18197.

Marshall, H., Studer, M., Pöpperl, H., Aparicio, S., Kuroiwa, A., Brenner, S., and Krumlauf, R. (1994). A conserved retinoic acid response element required for early expression of the homeobox gene *Hoxb-1*. Nature *370*, 567–571.

McClintock, J.M., Carlson, R., Mann, D.M., and Prince, V.E. (2001). Consequences of Hox gene duplication in the vertebrates: an investigation of the zebrafish Hox paralogue group 1 genes. Development *128*, 2471–2484.

McClintock, J.M., Kheirbek, M.A., and Prince, V.E. (2002). Knockdown of duplicated zebrafish hoxb1 genes reveals distinct roles in hindbrain patterning and a novel mechanism of duplicate gene retention. Development *129*, 2339–2354.

Megason, S.G. (2009). In toto imaging of embryogenesis with confocal time-lapse microscopy. Methods Mol. Biol. *546*, 317–332.

Moore, S., Ribes, V., Terriente, J., Wilkinson, D., Relaix, F., and Briscoe, J. (2013). Distinct regulatory mechanisms act to establish and maintain Pax3 expression in the developing neural tube. PLoS Genet. *9*, e1003811.

Ninov, N., Borius, M., and Stainier, D.Y. (2012). Different levels of Notch signaling regulate quiescence, renewal and differentiation in pancreatic endocrine progenitors. Development *139*, 1557–1567.

Oxtoby, E., and Jowett, T. (1993). Cloning of the zebrafish *krox-20* gene (*krx-20*) and its expression during hindbrain development. Nucleic Acids Res. *21*, 1087–1095.

Parker, H.J., and Krumlauf, R. (2017). Segmental arithmetic: summing up the Hox gene regulatory network for hindbrain development in chordates. Wiley Interdiscip. Rev. Dev. Biol. *6*, e286.

Parslow, A., Cardona, A., and Bryson-Richardson, R.J. (2014). Sample drift correction following 4D confocal time-lapse imaging. J. Vis. Exp. e51086, https://doi.org/10.3791/51086.

Pasini, A., and Wilkinson, D.G. (2002). Stabilizing the regionalisation of the developing vertebrate central nervous system. Bioessays *24*, 427–438.

Prince, V.E., Moens, C.B., Kimmel, C.B., and Ho, R.K. (1998). Zebrafish *hox* genes: expression in the hindbrain region of wild-type and mutants of the segmentation gene, *valentino*. Development *125*, 393–406.

Robu, M.E., Larson, J.D., Nasevicius, A., Beiraghi, S., Brenner, C., Farber, S.A., and Ekker, S.C. (2007). p53 activation by knockdown technologies. PLoS Genet. *3*, e78.

Rydeen, A., Voisin, N., D'Aniello, E., Ravisankar, P., Devignes, C.S., and Waxman, J.S. (2015). Excessive feedback of Cyp26a1 promotes cell non-autonomous loss of retinoic acid signaling. Dev. Biol. *405*, 47–55.

Rydeen, A.B., and Waxman, J.S. (2014). Cyp26 enzymes are required to balance the cardiac and vascular lineages within the anterior lateral plate mesoderm. Development *141*, 1638–1648.

Schilling, T.F., Prince, V., and Ingham, P.W. (2001). Plasticity in zebrafish hox expression in the hindbrain and cranial neural crest. Dev. Biol. *231*, 201–216.

Schilling, T.F., Sosnik, J., and Nie, Q. (2016). Visualizing retinoic acid morphogen gradients. Methods Cell Biol. *133*, 139–163.

Schneider, C.A., Rasband, W.S., and Eliceiri, K.W. (2012). NIH Image to ImageJ: 25 years of image analysis. Nat. Methods *9*, 671–675.

Schneider-Maunoury, S., Topilko, P., Seitanidou, T., Levi, G., Cohen-Tannoudji, M., Pournin, S., Babinet, C., and Charnay, P. (1993). Disruption of Krox-20 results in alteration of rhombomeres 3 and 5 in the developing hindbrain. Cell *75*, 1199–1214.

Sela-Donenfeld, D., Kayam, G., and Wilkinson, D.G. (2009). Boundary cells regulate a switch in the expression of FGF3 in hindbrain rhombomeres. BMC Dev. Biol. *9*, 16.

Sevetson, B.R., Svaren, J., and Milbrandt, J. (2000). A novel activation function for NAB proteins in EGR-dependent transcription of the luteinizing hormone beta gene. J. Biol. Chem. *275*, 9749–9757.

Sham, M.H., Vesque, C., Nonchev, S., Marshall, H., Frain, M., Das Gupta, R., Whiting, J., Wilkinson, D., Charnay, P., and Krumlauf, R. (1993). The zinc finger gene *Krox-20* regulates *Hoxb-2 (Hox2.8)* during hindbrain segmentation. Cell *72*, 183–196.

Shimozono, S., Iimura, T., Kitaguchi, T., Higashijima, S., and Miyawaki, A. (2013). Visualization of an endogenous retinoic acid gradient across embryonic development. Nature *496*, 363–366.

Sirbu, I.O., Gresh, L., Barra, J., and Duester, G. (2005). Shifting boundaries of retinoic acid activity control hindbrain segmental gene expression. Development *132*, 2611–2622.

Sosnik, J., Zheng, L., Rackauckas, C.V., Digman, M., Gratton, E., Nie, Q., and Schilling, T.F. (2016). Noise modulation in retinoic acid signaling sharpens segmental boundaries of gene expression in the embryonic zebrafish hindbrain. Elife *5*, e14034.

Standley, H.J., Zorn, A.M., and Gurdon, J.B. (2001). eFGF and its mode of action in the community effect during *Xenopus* myogenesis. Development *128*, 1347–1357.

Studer, M., Pöpperl, H., Marshall, H., Kuroiwa, A., and Krumlauf, R. (1994). Role of a conserved retinoic acid response element in rhombomere restriction of Hoxb-1. Science *265*, 1728–1732.

Taylor, H.B., Khuong, A., Wu, Z., Xu, Q., Morley, R., Gregory, L., Poliakov, A., Taylor, W.R., and Wilkinson, D.G. (2017). Cell segregation and border sharpening by Eph receptor-ephrin-mediated heterotypic repulsion. J. R. Soc. Interface *14*, 20170338.

Terriente, J., Gerety, S.S., Watanabe-Asaka, T., Gonzalez-Quevedo, R., and Wilkinson, D.G. (2012). Signalling from hindbrain boundaries regulates neuronal clustering that patterns neurogenesis. Development *139*, 2978–2987.

Theil, T., Frain, M., Gilardi-Hebenstreit, P., Flenniken, A., Charnay, P., and Wilkinson, D.G. (1998). Segmental expression of the EphA4 (Sek-1) receptor tyrosine kinase in the hindbrain is under direct transcriptional control of Krox-20. Development *125*, 443–452.

Trainor, P., and Krumlauf, R. (2000). Plasticity in mouse neural crest cells reveals a new patterning role for cranial mesoderm. Nat. Cell. Biol. *2*, 96–102.

Tuazon, F.B., and Mullins, M.C. (2015). Temporally coordinated signals progressively pattern the anteroposterior and dorsoventral body axes. Semin. Cell Dev. Biol. *42*, 118–133.

Tumpel, S., Wiedemann, L.M., and Krumlauf, R. (2009). Hox genes and segmentation of the vertebrate hindbrain. Curr. Top. Dev. Biol. *88*, 103–137.

Voiculescu, O., Taillebourg, E., Pujades, C., Kress, C., Buart, S., Charnay, P., and Schneider-Maunoury, S. (2001). Hindbrain patterning: *Krox20* couples segmentation and specification of regional identity. Development *128*, 4967–4978.

Wang, Q.X., Holmes, W.R., Sosnik, J., Schilling, T., and Nie, Q. (2017). Cell sorting and noise-induced cell plasticity coordinate to sharpen boundaries between gene expression domains. PLoS Comput. Biol. *13*, e1005307.

Weicksel, S.E., Gupta, A., Zannino, D.A., Wolfe, S.A., and Sagerstrom, C.G. (2014). Targeted germ line disruptions reveal general and species-specific roles for paralog group 1 hox genes in zebrafish. BMC Dev. Biol. *14*, 25.

Westerfield, M. (2007). The Zebrafish Book (University of Oregon).

White, R.J., Nie, Q., Lander, A.D., and Schilling, T.F. (2007). Complex regulation of cyp26a1 creates a robust retinoic acid gradient in the zebrafish embryo. PLoS Biol. *5*, e304.

White, R.J., and Schilling, T.F. (2008). How degrading: Cyp26s in hindbrain development. Dev. Dyn. *237*, 2775–2790.

Xu, Q., Alldus, G., Holder, N., and Wilkinson, D.G. (1995). Expression of truncated Sek-1 receptor tyrosine kinase disrupts the segmental restriction of gene expression in the *Xenopus* and zebrafish hindbrain. Development *121*, 4005–4016.

Xu, Q., Holder, N., Patient, R., and Wilson, S.W. (1994). Spatially regulated expression of three receptor tyrosine kinase genes during gastrulation in the zebrafish. Development *120*, 287–299.

Xu, Q., Mellitzer, G., Robinson, V., and Wilkinson, D.G. (1999). In vivo cell sorting in complementary segmental domains mediated by Eph receptors and ephrins. Nature *399*, 267–271.

Zhang, L., Radtke, K., Zheng, L., Cai, A.Q., Schilling, T.F., and Nie, Q. (2012). Noise drives sharpening of gene expression boundaries in the zebrafish hindbrain. Mol. Syst. Biol. *8*, 613.

Zhao, Q.S., Dobbs-McAuliffe, B., and Linney, E. (2005). Expression of cyp26b1 during zebrafish early development. Gene Expr. Patterns *5*, 363–369.

Zigman, M., Laumann-Lipp, N., Titus, T., Postlethwait, J., and Moens, C.B. (2014). Hoxb1b controls oriented cell division, cell shape and microtubule dynamics in neural tube morphogenesis. Development *141*, 639–649.

STAR★METHODS

KEY RESOURCES TABLE

REAGENT or RESOURCE	SOURCE	IDENTIFIER
Antibodies		
Anti-Digoxigenin-AP (*in situ*)	Roche	Cat# 11093274910; RRID: AB_514497
Anti-Fluorescein-AP (*in situ*)	Roche	Cat# 11426338910; RRID: AB_514504
Anti-GFP (IF)	Torrey Pines Biolabs	Cat# TP401 071519; RRID: AB_10013661
Anti-Myc (IF)	Santa Cruz Biotechnology	Cat# sc-40; RRID: AB_627268
Anti-Rabbit Alexa Fluor 488 (IF)	Thermo Fisher Scientific	Cat# R37116; RRID: AB_2556544
Anti-Mouse Alexa Fluor 488 (IF)	Thermo Fisher Scientific	Cat# R37120; RRID: AB_2556548
Chemicals, Peptides, and Recombinant Proteins		
BCIP	Roche	Cat# 11383221001
NBT	Roche	Cat# 11383213001
AGN193109	Santa Cruz Biotechnology	Cat# sc-210768
Fast Blue BB	Sigma	Cat# F3378
NAMP (Naphthol AS-MX phosphate)	Sigma	Cat# N5000
Fast Red TR/Naphthol AS-MX Tablets	Sigma	Cat# F4648
Cas9 NLS	New England Biolabs	Cat# M0646M
Experimental Models: Organisms/Strains		
Zebrafish *Danio rerio*: WT	N/A	N/A
Zebrafish *Danio rerio*: Tg [egr2b:H2B-Citrine]	This study	N/A
Zebrafish *Danio rerio*: Tg [CNE1:egr2b-Myc]	This study	N/A
Zebrafish *Danio rerio*: Tg [HS:egr2b-Myc]	This study	N/A
Oligonucleotides		
See supplementary table	This study	N/A
Recombinant DNA		
pCS2b1a: probe synthesis hoxb1a: linearize KpnI: polymerase T3	McClintock et al., 2001	N/A
pBS-hoxa2: probe synthesis hoxa2: linearize KpnI: polymerase T3	Prince et al., 1998	N/A
pCS2-cyp26b1: probe synthesis cyp26b1: linearize BglII: polymerase Sp6	Hernandez et al., 2007	N/A
pCS2-cyp26c1: probe synthesis cyp26c1: linearize SmaI: polymerase T7	Hernandez et al., 2007	N/A
pBS-krox-20: full-length probe synthesis egr2b: linearize PstI: polymerase: T3	Oxtoby & Jowett, 1993	N/A
pBS-krox-20: 3′ UTR probe synthesis egr2b: linearize SphI: polymerase: T3	Oxtoby & Jowett, 1993	N/A
pT3Ts-Tol2: mRNA synthesis Tol2: linearize SpeI: polymerase T3	Balciunas et al., 2006	N/A
pDONR221	Thermo Fisher Scientific	Cat# 12536017
p5E-hsp70l	Kwan et al., 2007	N/A
p3E-MTpA	Kwan et al., 2007	N/A
pDestTol2pACryGFP	Berger and Currie, 2013	N/A
pCS2-H2B-citrine: probe synthesis citrine: linearize XmaI: polymerase T3	Megason, 2009	N/A
pCS2-H2B-EGFP: mRNA synthesis H2B-eGFP: linearize NotI: polymerase Sp6	Megason, 2009	N/A
pMiniTol2-Pax3CNE1-TKprom-Gal4-UAS:H2Bcitrine	Moore et al., 2013	N/A

(*Continued on next page*)

Continued		
REAGENT or RESOURCE	SOURCE	IDENTIFIER
pDR274: linearize BsaI-HF	Hwang et al., 2013	N/A
MLM3613: mRNA synthesis cas9: linearize PmeI: polymerase T7	Hwang et al., 2013	N/A
Software and Algorithms		
FIJI (ImageJ)	Schneider et al., 2012	N/A
Fluorender	N/A	N/A

CONTACT FOR REAGENT AND RESOURCE SHARING

Further information and requests for resources and reagents should be directed to and will be fulfilled by the Lead Contact, David G. Wilkinson (david.wilkinson@crick.ac.uk).

EXPERIMENTAL MODEL AND SUBJECT DETAILS

Maintenance of Zebrafish Strains

Wild type and transgenic zebrafish embryos were obtained by natural spawning and raised at 28.5°C as described (Westerfield, 2007). Embryos were staged by hours post-fertilization (hpf) at 28.5°C and/or morphological features (Kimmel et al., 1995). The zebrafish work was carried out under a UK Home Office Licence under the Animals (Scientific Procedures) Act 1986 and underwent full ethical review.

METHOD DETAILS

Constructs and Transgenesis

pMiniTol2-Pax3CNE1-TKprom-Gal4-UAS:Egr2b-Myc (referred to as CNE1:egr2b-Myc) was created by replacing the H2B-citrine coding sequence from pMiniTol2-Pax3CNE1-TKprom-Gal4-UAS:H2B-citrine (Moore et al., 2013) with the *egr2b* coding sequence and C-terminal Myc tag, amplified from hsp70:Egr2b-Myc-ACG. For transient transgenesis, 15-18 pg DNA and 36 pg Tol2 transposase RNA were injected into one-cell stage embryos.

The HS:egr2b-Myc;αCrystallin-GFP expression vector was generated using plasmids from the Tol2Kit (Kwan et al., 2007) and pDestTol2pACryGFP destination vector (Addgene plasmid #64022) (Berger and Currie, 2013). The *egr2b* middle entry vector (pME-*egr2b*) was created by BP recombination between the pDONR-221 vector and the *egr2b* coding sequence (Oxtoby and Jowett, 1993) flanked by attB sites. The mBait-H2B-Citrine donor plasmid was generated by insertion of a cFos minimal promoter, mBait gRNA target site, H2B-Citrine coding sequence and SV40 polyadenylation signal into a pBluescript II KS backbone.

The Tg[egr2b:H2B-Citrine] line (fci3) was generated by CRISPR/Cas9-mediated insertion using the strategy described in (Kimura et al., 2014). Embryos were injected at the one cell stage with Cas9 mRNA (350 pg), mBait gRNA (50 pg), gRNA (100 pg) targeting a sequence 893 bp upstream of the transcriptional start site of Egr2b (ATTCTGAGCTATCCAGTACGG), and mBait-H2B-Citrine donor plasmid (10-20 pg). CRISPR/Cas9-mediated mutation of the ephA4 and cyp26c1 genes was carried out using the gRNAs described in Table S1. The hs-egr2b line (fci4) was generated by injection of an Hsp70:Egr2b-Myc;αCrystallin-GFP expression vector.

Morpholino Injection

Morpholino oligonucleotides (MOs) were obtained from GeneTools (Oregon, USA) and were aliquoted and stored at room temperature in glass vials. MOs were injected into the yolk of blastomeres at the 1-4 cell stage in combination with a p53 translation-blocking MO to block off–target effects mediated by activation of apoptotic pathways (Gerety and Wilkinson, 2011; Robu et al., 2007). 4 ng MO was injected, except for ephA4 MO for which 5 ng was used. The MOs used are as follows: egr2b translation-blocking MO (AGTTT TAGCTGTCATCGTGAAGTCC); egr2a translation-blocking MO (CATGTGCTCCATGTTGGGAAGATTT); ephA4 (Cooke et al., 2005); cyp26b1 and cyp26c1 (Hernandez et al., 2007); hoxb1a and hoxb1b (McClintock et al., 2002); p53 (Langheinrich et al., 2002). Control morphant embryos were injected with a corresponding amount of the standard control MO (GeneTools). egr2a and egr2b MO block egr2b autoregulation and cause loss of r3 and r5 as occurs in an egr2b zebrafish mutant (Bouchoucha et al., 2013) and egr2 mouse mutant (Schneider-Maunoury et al., 1993; Voiculescu et al., 2001). All other MOs used have previously been characterized and their efficacy and specificity demonstrated as follows. ephA4 MO blocks generation of EphA4 protein and disrupts the sharpening of the r2/r3, r3/r4 and r5/r6 borders (Cooke et al., 2005; Terriente et al., 2012); we show that the same phenotype occurs in an ephA4 mutant (fci2) which has a 4 bp deletion at residue 158 in the ligand-binding domain (Figure S1). cyp26b1 MO and cyp26c1 MO cause posteriorisation of the hindbrain when combined with a cyp26a1 mutant, and this is phenocopied by chemical blocking of Cyp26 enzymes (Hernandez et al., 2007). We show that the phenotype of ectopic egr2-expressing cells seen in morphants also occurs in cyp26c1 mutant embryos (Figure S3). hoxb1a MO disrupts hoxb1a autoregulation, and hoxb1b MO causes a decreased size of r4 and increase in r3 (McClintock et al., 2002); the same phenotypes but more severe are seen in hoxb1a and hoxb1b mutants

(Weicksel et al., 2014; Zigman et al., 2014). p53 MO blocks activation of apoptotic pathways, as also occurs in p53 mutant zebrafish (Gerety and Wilkinson, 2011; Langheinrich et al., 2002; Robu et al., 2007).

Pharmacological and Heat Shock Treatments

To block RA signaling, dechorionated embryos were treated with 10 μM AGN193109 in 2% DMSO in 0.65x Danieau's solution; control embryos were treated with 2% DMSO in 0.65x Danieau's solution. For induction of heat shock regulated constructs, embryos were placed at 36°C for 30 min in pre-warmed 0.65 x Danieau's solution at the stages indicated.

Cell Transplantation

Donor embryos were injected at the 1 cell stage with 100 pg H2B-eGFP RNA. Cell transplantation was performed at 4 hpf, with donor cells targeted to the future hindbrain based on fate maps (Kimelman and Martin, 2012).

In Situ Hybridization and Immunocytochemistry

Embryos of the desired stage were fixed in 4% paraformaldehyde/PBS for 3 h at room temperature or overnight at 4°C. Fixed embryos were stored in 100% methanol at −20°C prior to processing by *in situ* hybridization or immunocytochemistry. Probes used for *in situ* hybridization have been previously described: egr2b (Oxtoby and Jowett, 1993); citrine (Megason, 2009); hoxb1a (McClintock et al., 2001); cyp26b1 (Zhao et al., 2005); cyp26c1 (formerly known as cyp26d1) (Gu et al., 2005). Antisense riboprobes were labeled with digoxigenin-UTP or fluorescein-UTP. *In situ* hybridization and color development with BCIP/NBT or Fast Blue was conducted as previously described (Lauter et al., 2011; Xu et al., 1994). Two color fluorescent *in situ* hybridization was carried out using Fast Blue and Fast Red substrates. After BCIP/NBT color development, embryos were re-fixed, cleared in 70% glycerol/PBS, and mounted for imaging using a Zeiss Axioplan2 with Axiocam HRc camera. In some experiments (Figures 6I–6M), egr2b transcripts were detected by hybridization chain reaction (HCR) using the protocol detailed in (Choi et al., 2016). A kit containing a DNA probe set, a DNA HCR amplifier, and hybridization, wash and amplification buffers was purchased from Molecular Instruments (molecularinstruments.org). The egr2b probes initiate B5 (Alexa594) amplifier.

For immunocytochemistry, embryos were fixed for 2 h at room temperature in 4% paraformaldehyde, washed in PBT, then dechorionated and blocked for 1 h in 5% goat serum in PBT. Primary antibodies were used at the following concentrations in 2.5% goat serum: rabbit anti-GFP (1:500; 1:400 after *in situ* hybridization); Myc (1:400 after *in situ* hybridization). Secondary antibodies were used at the following concentrations: goat anti-rabbit Alexa Fluor® 488 (IgG H + L) (1:500 dilution); goat anti-mouse Alexa Fluor® 488 (IgG H + L) (1:500 dilution). Embryos were incubated in DAPI to stain nuclei. Embryos were then cleared in 70% glycerol/PBS, and mounted for imaging on a Leica TCS SP2 confocal microscope or Zeiss LSM700 confocal microscope.

Live Imaging and Analysis

Dechorionated embryos were embedded in 0.6% low melting agarose (Sigma)/0.5x Danieau's solution within a 1% agarose (Bio-Rad Laboratories Inc.)/0.5x Danieau's solution-coated chambered coverslip with individual embryo-shaped wells filled with 0.5x Danieau's solution. Embryos were imaged using an inverted Zeiss LSM710 confocal microscope with 20x lens, NA 0.8. Z-stacks with a slice depth of 2 μm were acquired every 3 minutes. ImageJ (NIH) was used for image processing. The Correct 3D Drift ImageJ plug-in (Parslow et al., 2014) was used to correct for 3D drift in time lapse movies. FluoRender v. 2.20.0 (University of Utah) was used to create 3D projections.

QUANTIFICATION AND STATISTICAL ANALYSIS

Quantification of Ectopic Cells

The number of cells in r2, r4 and r6 that express egr2b transcripts and/or H2B-citrine reporter was manually counted from confocal stacks. Statistical significance was determined using Welsh's T test in GraphPad Prism 7 as indicated in the Figure legends.

Developmental Cell, Volume *45*

Supplemental Information

Cell Identity Switching Regulated by Retinoic Acid Signaling Maintains Homogeneous Segments in the Hindbrain

Megan Addison, Qiling Xu, Jordi Cayuso, and David G. Wilkinson

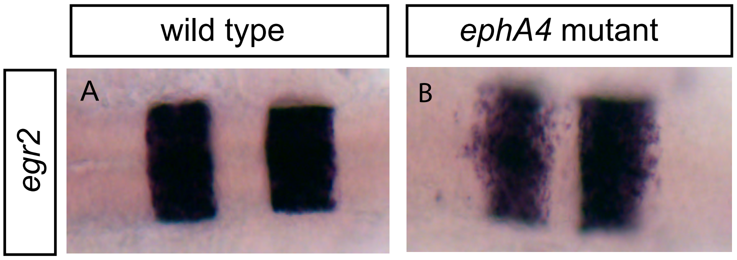

Figure S1, related to Figure 3. Analysis of *ephA4* mutant embryos
(A, B): An *ephA4* mutant has disrupted sharpening of the r2/r3, r3/r4 and r5/r6 borders. MO-mediated knockdown disrupts *ephA4* function since the same phenotype occurs in *ephA4* morphant embryos (Cooke et al., 2005; Terriente et al., 2012).

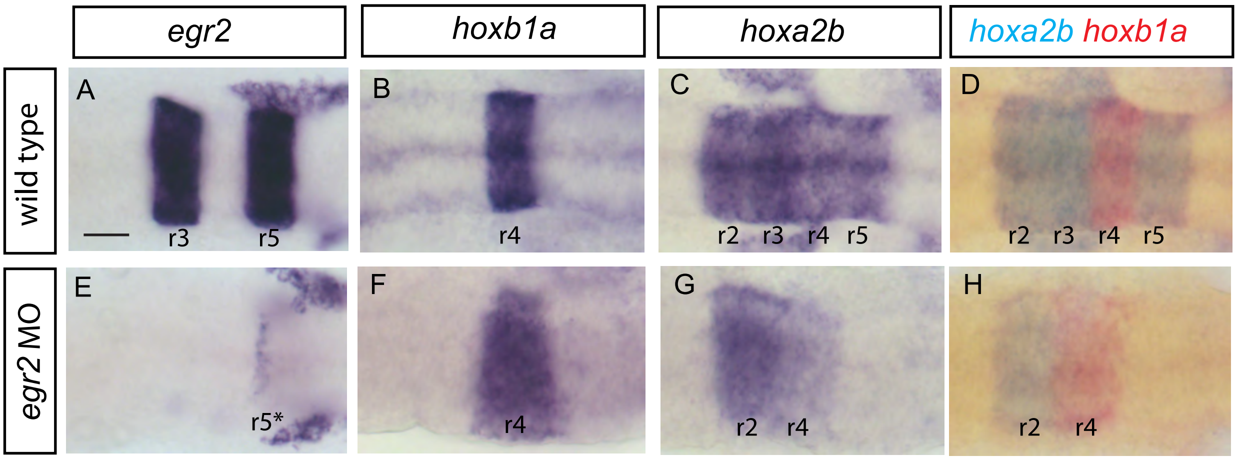

Figure S2, related to Figure 4. Analysis of *egr2* knockdown embryos
(A-H): Knockdown of *egr2a* and *egr2b* results in loss of *egr2b* expression in r3 and severe reduction of expression in r5* at 17 hpf (E) compared to control embryos (A). Following loss of r3 territory, the flanking segments still express *hox* genes that mark r2 (*hoxa2*) and r4 (*hoxb1* plus *hoxa2*): compare controls (B-D) with *egr2* knockdown embryos (F-H). A-C and E-G are single in situ hybridizations, and D, H are double in situ hybridizations, as indicated. Scale bar: 50 μm.

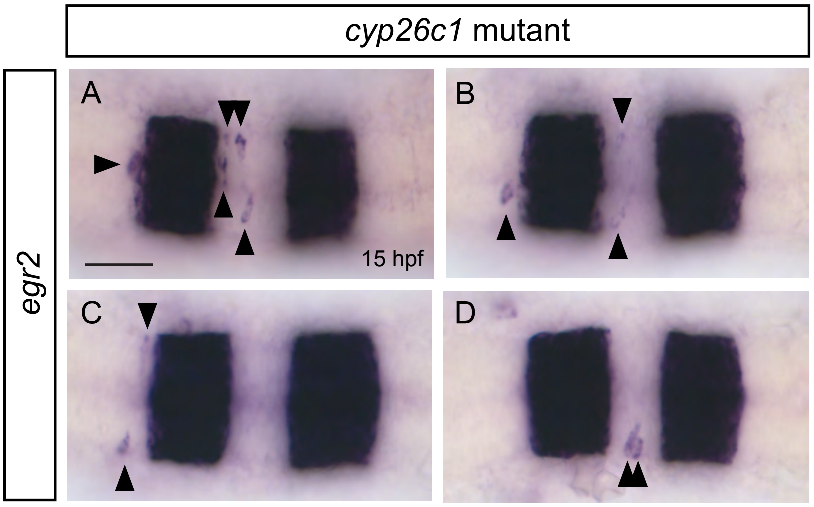

Figure S3, related to Figure 6A-E. *cyp26c1* mutant embryos have ectopic *egr2*-expressing cells (A-D): Expression of *egr2* was analyzed in *cyp26c1* mutant embryos. Ectopic *egr2*-expressing cells were observed in r2 and r4 (arrowheads), as seen in morphants (Fig.6B). That this phenotype occurs following inactivation of *cyp26c1* alone suggests that RA levels in r2 and r4 are altered sufficiently to disrupt identity switching. Scale bar: 50 µm.

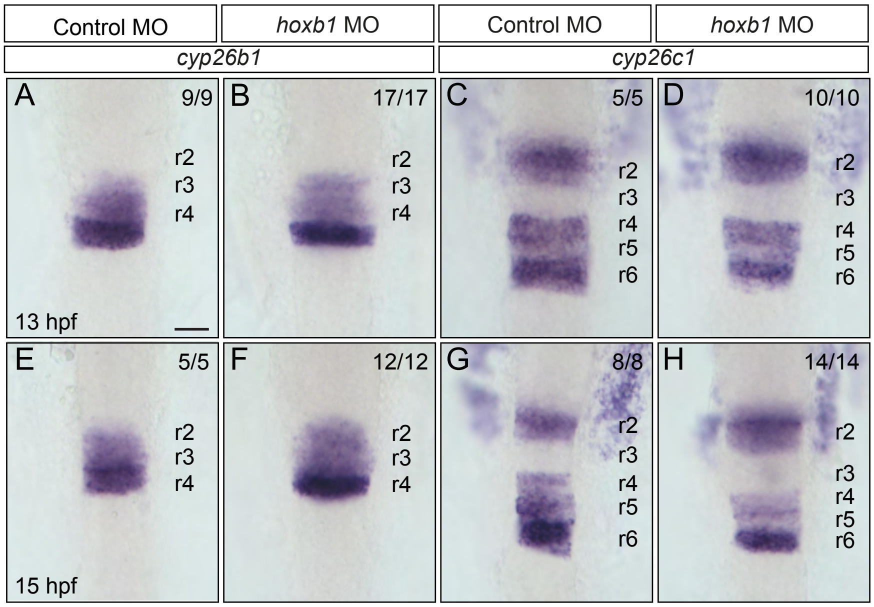

Figure S4, related to Figure 6F-H. *hoxb1* knockdown does not alter *cyp26b1* or *cyp26c1* expression
(A-H): *hoxb1a* and *hoxb1b* were knocked down and expression of *cyp26b1* and *cyp26c1* analyzed. While knockdown of *hoxb1* increases the size of r3 at the expense of r4, *cyp26b1* is still highly expressed in r4 at 13 hpf (B) and 15 hpf (E), as in control embryos (A, E). Similarly, *cyp26c1* is still expressed in r4 of *hoxb1* morphants at 13 hpf (D) and 15 hpf (H), as in control embryos (C, G). Embryos in are flat-mounted with anterior to the top. Scale bars: 50 μm.

Table S1. Related to STAR Methods section. Oligonucleotides used in the paper

Cloning oligos

Name	Sequence 5'-3'	Target
egr2b-attB F	GGGGACAAGTTTGTACAAAAAAGCAGGCTGGACTTCACGATGACAGCTAAAACTTTG	egr2b for Gateway pME
egr2b-attB R	GGGGACCACTTTGTACAAGAAAGCTGGGTGGTTTGAACTGGACGAGCAGATGC	egr2b for Gateway pME
egr2b-Myc Amp F	GATCGTCGACGCTGGACTTCACGATGACA	egr2b-Myc for CNE1:egr2b-Myc
egr2b-Myc Amp R	GGATCATCATCGATGGTAC	egr2b-Myc for CNE1:egr2b-Myc
mBait insertion F	AGCGCGGCTGCTGCGGTTCCAGAGGTGGATCGATCTCGAGAAGCTTGACGT	annealed
mBait insertion R	CAAGCTTCTCGAGATCGATCCACCTCTGGAACCGCAGCAGCCGCGCT	annealed
H2B-Citrine-pA F	GATCAAGCTTCTGCAGTCGACGGTACCGCCACC	H2B-Citrine-polyA
H2B-Citrine-pA R	CGCCGCGGCCGCGAATTAAAAAACCTCCCACAC	H2B-Citrine-polyA
cFos insertion F	CGATCCAGTGACGTAGGAAGTCCATCCATTCACAGCGCTTCTATAAAGGCGCCAGCTGA GGCGCCTACTACTCCAACCGCGACTGCAGCGAGCAACTA	annealed
cFos insertion R	AGCTTAGTTGCTCGCTGCAGTCGCGGTTGGAGTAGTAGGCGCCTCAGCTGGCGCCTTTATAGA AGCGCTGTGAATGGATGGACTTCCTACGTCACTGGAT	annealed

Morpholinos

Name	Sequence 5'-3'	Target	Reference	Dose
Standard control MO	CCTCTTACCTCAGTTACAATTTATA			4-5 ng/embryo
p53 MO	GCGCCATTGCTTTGCAAGAATTG	p53	Langheinrich et al., 2002	4 ng/embryo
egr2b MO	AGTTTTAGCTGTCATCGTGAAGTCC	egr2b	this study	4 ng/embryo
egr2a MO	CATGTGCTCCATGTTGGGAAGATTT	egr2a	this study	4 ng/embryo
ephA4 MO	AACACAAGCGCAGCCATTGGTGTC	ephA4	Cooke et al., 2005	5 ng/embryo
cyp26b1 MO	CTCGAAGAGCATGGCTGTGAACGTC	cyp26b1	Hernandez et al., 2007	4 ng/embryo

cyp26c1 MO	AAACTCGGTTATCCTCACCTTGCGC	cyp26c1	Hernandez et al., 2007	4 ng/embryo
hoxb1a MO	GGAACTGTCCATACGCAATTAA	hoxb1a	McClintock et al., 2002	4 ng/embryo
hoxb1b MO	AATTCATTGTTGACTGACCAAGCAA	hoxb1b	McClintock et al., 2002	4 ng/embryo

gRNAs

Name	Sequence 5'-3'	Target	Reference	Dose
egr2b	(GG)ATTCTGAGCTATCCAGTACGG	egr2b	this study	10 pg/embryo
mBait	GGCTGCTGCGGTTCCAGAGGTGG	mBait	this study	50 pg/embryo
cyp26c1 1	CCATGGATCCCTGCGGGAGTGGG	cyp26c1	this study	32 pg/embryo
cyp26c1 2	GGCCAGCCCATGGATCCCTGCGG	cyp26c1	this study	32 pg/embryo
ephA4	CCTGCGTGAAGCTTTCATCAGCC	ephA4	this study	32 pg/embryo

gRNA oligos

Name	Sequence 5'-3'	Target
egr2b F	TAGGATTCTGAGCTATCCAGTA	egr2b
egr2b R	AAACTACTGGATAGCTCAGAAT	egr2b
mBait F	TAGGCTGCTGCGGTTCCAGAGG	mBait
mBait R	AAACCCTCTGGAACCGCAGCAG	mBait

Target sequences for HCR detection of egr2b transcripts

Sequences 5'-3'	Target	Reference
CTGACAGCTTTCCACATGTAACGCTTCGTGCGCGCGTTCGCACAGACACAAC<br>ATTCTGTGAACATCCGAGCGAGTGCTTCTTAGGACTTCACGATGACAGCTAA<br>ACGGGGATATGAGCACGGAGAAGCGCGCCCTCGACTTAGCCTACTCCAGCAG<br>GACCAGTGCCTGACGGACCCCGGCTACCCACAGCTTACACTCCGCAGAATTT<br>GCATCTATTCGGTGGACGAGCTTGCCACAACACTGCCAGCCTCTGTGACTAT<br>ATAACGATTTAGGAGGACATTACGAGCAGATAAACGCAGGAGATGGCCTGAT<br>TCGCGCAACCAGCTGGCCCTCGCAACCAAACTTTTACCTACATGGGAAAGTT<br>CCATCGACTCCCAGTACCCGGGAAACTGGAACCCAGAGGGCGTGATCAACAT<br>AGTCGCATGCTGACTGACCCAAACGCACTTTGTTTGAATATTTAGGCCAATT<br>CAAACTAAACTTTCAAGGAGGTGTCTAACATCACTATTTTCTCCCATCGTCG<br>ACTGTTATACATGTATTTGTTATGAAGTGACTAACGCGGGTCAATAATGTCC<br>CTTTGGAGAAAGCCCCTGTGAGTCTCGGTGGCTTTGTGCACCCTCTTGCCGA<br>TCTCATATCCTCCGCCATCCTACTCCTCTCCAAAGCCAAACGCCGACTCTGG<br>TGTTCCCTATAATCCCGGACTACGCCGGCTTTTTCCAACCTCCGTGCCAGAG<br>AATTACCGCCTCCTCTAACCCCCCTGAACACTATCAGGAACTTCACGCTAGG<br>AGTTCGCGAGGAGCGACGAAAGAAAGAGACACACCAAAATCCACCTGCGACA<br>AAGAGCGAAAGTCCTCCTCGTCGTCCACAGGAGTGTCCAGCTCAGAGCGCGG<br>TCGCCACGAGCATCTGCTCGTCCAGTTCAAACCAGTGAACTTTCAACTGGAC<br>TGCAATATCCAACACTATGTAGGCAATACAATAATGCACCGTTCGTGTTTTA<br>AAAACTGGGTGGACTGATGAGGTGAAATCAAACCACAGTGCCAAACATGGAC	egr2b	this study
